# Respectful care during childbirth and educational inequities: a cross-sectional survey using a multidomain index across four hospitals in Malawi

**DOI:** 10.1136/bmjopen-2026-118574

**Published:** 2026-10-21

**Authors:** Bianca Kandeya, Claudia Hanson, Soha El Halabi, Kristi Sidney Annerstedt, William Stones, Effie Chipeta, Sunjuri Sun

**Affiliations:** 1 Centre for Reproductive Health, Kamuzu University of Health Sciences, Blantyre, Southern Region, Malawi; 2 Department of Global Public Health, Karolinska Institute, Stockholm, Stockholm County, Sweden; 3 Department of Health Systems and Policy, Kamuzu University of Health Sciences, Blantyre, Southern Region, Malawi

**Keywords:** Attitude, Burnout, Professional, Cross-Sectional Studies, Decision Making, Public Hospitals

## Abstract

**Abstract:**

**Objective:**

This study aimed to apply a validated multidomain respectful maternity care index to assess women’s reported childbirth care experiences across four hospitals in Malawi, and to examine how reported less respectful maternity care varied by maternal education.

**Design:**

Cross-sectional survey nested within the ALERT implementation research programme. Data were analysed using descriptive statistics and logistic regression, with sensitivity analyses using continuous outcome, modified Poisson and narrower abuse-focused outcome models.

**Setting:**

The study was conducted in four high-volume secondary-level maternity units in Malawi’s Central Region, including three public facilities and one faith-based facility.

**Participants:**

1004 postpartum women who gave birth in participating hospitals to infants weighing at least 1000 g or at a gestational age of at least 28 weeks. Data were collected between December 2021 and March 2024 across five survey rounds.

**Primary outcome measures:**

The primary outcome was reported less respectful maternity care, measured using a validated composite score covering absence of abuse, privacy and confidentiality, dignity and respect, communication and supportive care, social support and the hospital environment. Scores were expressed as a percentage of best possible care and dichotomised at the 25th percentile (≤84.5/100). Individual respectful care indicators, including verbal abuse, privacy violations and non-consented care, were also assessed, alongside variations in reported care experiences across maternal educational levels.

**Results:**

Overall, 16% of women were classified as reporting less respectful maternity care based on the composite score. Verbal abuse was the most frequently reported form of mistreatment, while non-consented care, neglect and privacy violations were less common; physical abuse and bribery were rare. Women with secondary or higher education had higher odds of reporting less respectful maternity care than women with primary or no education (adjusted OR 1.48, 95% CI 1.02 to 2.15; p=0.04) defined as being in the lowest quartile of respectful maternity care scores. Sensitivity analyses using the continuous respectful maternity care score and modified Poisson regression yielded similar evidence of an association with education, whereas no association was observed for the narrower abuse-focused outcome.

**Conclusion:**

Less respectful maternity care remains a concern in Malawi. Women with secondary or higher education had higher odds of reporting poorer respectful maternity care. This association may reflect differences in expectations, recognition, reporting or true experiences of care, which could not be distinguished in this study. Research should examine education-related reporting differences, while reforms strengthen communication, accountability and maternity infrastructure.

**STRENGTHS AND LIMITATIONS OF THIS STUDY**

- This study used a large, prospectively collected, multifacility sample of postpartum women across four hospitals in Malawi’s Central Region.
- Data were collected using structured questionnaires adapted from validated respectful and person-centred maternity care tools.
- Tablet-based Research Electronic Data Capture (REDCap) data collection with real-time validation checks supported standardised data capture and improved data quality.
- The cross-sectional design limits causal interpretation of associations between education and reported less respectful maternity care.
- The study was conducted in hospitals enrolled in the ALERT implementation research programme, which limits generalisability to routine maternity care settings in Malawi.

## Background

 Provision of quality maternal health services requires not only appropriate clinical care but also care that is respectful, dignified and free from abuse.^1 2^ The WHO defines mistreatment during childbirth as interactions or facility conditions that are disrespectful, abusive or neglectful, encompassing physical, verbal or emotional abuse; stigma and discrimination; non-consented care; breaches of privacy and confidentiality; poor communication; and health-system constraints that result in neglect or abandonment.^3–5^ Such experiences undermine women’s trust in the health system, discourage future facility use and compromise maternal and neonatal outcomes.^2 6 7^ Despite global progress in provision of quality maternal health, evidence from sub-Saharan Africa shows that violations of respectful maternity care remain widespread, with serious consequences for women’s physical and psychological well-being as well as their confidence in health services.^2 8 9^ Respectful maternity care, which emphasises dignity, privacy, autonomy and freedom from harm, is therefore central to ensuring quality and equity in maternal healthcare.^10 11^

Mistreatment during childbirth ranges from verbal abuse and non-consented care to abandonment and physical assault, remains widespread.^1 12^ These behaviours occur within overstretched health systems and are reinforced by hierarchical provider relationships, gendered power structures and limited accountability mechanisms.^13^ Evidence also suggests that young, poor, rural and less-educated women may be more exposed to mistreatment or less able to challenge or report it, not because they experience it less, but because structural inequities shape both vulnerability and voice.^12 14 15^

In Malawi, more than 90% of births now occur in health facilities, reflecting progress in access to skilled care; however, significant gaps remain in ensuring respectful maternity care.^16 17^ Women have reported being shouted at or scolded, threatened with physical harm, ignored when in need of help, denied privacy and subjected to non-consented procedures.^18 19^

Although the government has demonstrated commitment by adopting the WHO Respectful Maternity Care Charter, updating national maternal and newborn care guidelines and incorporating respectful care principles into midwifery training, implementation into routine practice remains inconsistent, reports of verbal abuse, neglect and breaches of autonomy continue to emerge across facilities despite these efforts.^19–21^

Socioeconomic factors, particularly a woman’s level of education, play a significant role in shaping childbirth experience, including the quality of interpersonal care a woman receives (communication, dignity, autonomy).^22 23^ Education also influences how women are perceived and treated by providers and their ability to advocate for themselves during care.^24^ Meta-analyses from sub-Saharan Africa further indicate that women with lower education or from poorer wealth indices are at heightened risk of mistreatment during facility-based childbirth.^25 26^ However, Malawi-specific evidence on how a mother’s education level specifically relates to her risk of mistreatment during childbirth remains scarce.

This study applies a validated multidomain respectful maternity care index to assess women’s reported childbirth care experiences across four hospitals in Malawi. Rather than focusing only on narrow indicators of abuse, the index captures broader dimensions of respectful and responsive care, including dignity, privacy, communication, social support, hospital environment and absence of physical and verbal abuse.

## Material and methods

### Study design

We used a cross-sectional study design to assess women’s reported respectful maternity care experiences and examined associations between education level and reported respectful maternity care during facility-based childbirth across four hospitals in Malawi’s Central Region. The research was nested within the larger four-country collaborative implementation research project Action Leveraging Evidence to Reduce Perinatal Mortality in Sub-Saharan Africa (ALERT), which aims to reduce perinatal mortality and improve experiences of care through strengthening the quality of routine intrapartum care.^27 28^ This study was reported in line with the Consensus-Based Checklist for Reporting of Survey Studies (CROSS) checklist for reporting of survey studies (see supplemental file 5).

### Study setting

Malawi, a low-income country in East Africa, faces persistent challenges in maternal health due to a low nurse-to-midwife density of 5.1 per 10 000 people and a maternal mortality ratio of 381 per 100 000 live births.^16 29^

We included three government hospitals, which provide free maternity care and one faith-based facility that operates on a fee-for-service basis.^30^ Nationally, about 60% of hospitals are publicly owned, while the remainder are mainly faith-based institutions under the Christian Health Association of Malawi, alongside a smaller number of private facilities that often serve rural or hard-to-reach areas.^31^ The purposive inclusion of both public and faith-based facilities allowed for comparison across different healthcare contexts.^32^

### Study tool

This hospital-based cross-sectional survey used validated items from Bohren’s mistreatment tool and Afulani’s person-centred maternity care scale to assess responsiveness, mistreatment during labour and birth and respectful newborn care, including breastfeeding.^33 34^ Data were collected in the immediate postnatal period, within 24 hours after vaginal birth and at least 48 hours after caesarean section. The questionnaire included 12 sections (≈120 items) covering demographics, childbirth costs, verbal and physical abuse, social support, provider communication, hospital environment, satisfaction and newborn care.

The tool was translated into Chichewa, Malawi’s predominant local language by bilingual nursing and public health experts. Forward translation by a native speaker was followed by cultural appropriateness, pilot testing during training and minor refinements. Supervisors ensured fidelity during data collection. Although formal back translation was not conducted, iterative refinement and supervision supported consistency and retention of meaning.

Psychometric validation of the tool using ALERT data from Benin, Malawi, Tanzania and Uganda produced factor-weighted scores for respectful treatment, healthcare responsiveness and an overall composite.^35^ Respectful treatment score included dignity, privacy, confidentiality and absence of abuse. Responsiveness score covered communication and supportive care, environment and social support. In this study, selected validated items were used to construct respectful care scores (see online supplemental file 1).

### Data collection

Data were collected across four Malawian hospitals within the ALERT stepped-wedge trial. Four trained data collectors (three female, one male; all with nursing/midwifery backgrounds) administered structured, pretested tablet-based Research Electronic Data Capture (REDCap) questionnaires hosted at Karolinska Institutet.^36 37^ Training covered administration, translation and ethics, with refreshers before each round. Linked to the perinatal e-registry, surveys required informed consent and benefited from strong standardisation and supervision from ALERT.

The ALERT intervention used a stepped-wedge design, with facilities transitioning from control to intervention at 6-month intervals. Recruitment and data collection ran from December 2021 to March 2024, aligning with intervention rollout. Approximately 50 postpartum women were recruited per facility every 6 months, yielding five rounds and ~1000 participants. Daily recruitment followed a randomised approach tailored to discharge volume: all eligible women were invited where <14 discharges; every second woman (1:2) was selected for 20–29 discharges; and every third woman (1:3) for ≥30 discharges. This ensured consistent and feasible sampling across sites. Facilities were visited sequentially, with data collection completed before intervention initiation. Women were classified as pre-intervention or post-intervention based on facility-specific crossover dates and their delivery or interview timing. Details of the crossover timing are provided in (online supplemental file 3).

Sample size was determined for the ALERT trial’s primary perinatal outcome, not respectful care examined in this analysis. It assumed a 75% reduction from a 4% baseline, intraclass correlation coefficient 0.1, 77% power and 1% outcome proportion.^27^ No separate power calculation assessed associations between maternal education and care experiences; thus, regression findings should be interpreted as exploratory and hypothesis-generating rather than definitive.

## Participants

Participants were eligible if they gave birth at study hospitals with birth weight ≥1000 g or gestational age ≥28 weeks and provided informed consent for participation and linkage to the ALERT perinatal e-registry.^27^ Data collectors screened registers daily, approached eligible women and obtained consent after explaining study aims, procedures, risks and benefits. Interviews were conducted privately in Chichewa using structured questionnaires on tablets with real-time validation checks, with data securely synced daily to central database, with deferred uploads during periods of poor connectivity.^36^

### Variables

Variables were derived by grouping questionnaire items into six domains: absence of physical and verbal abuse, privacy and confidentiality, respect and dignity, communication and supportive care, social support and hospital environment. For example, verbal abuse included shouting, mocking or threats, while physical abuse included acts such as slapping or pinching (table 1). A psychometrically validated scoring approach developed within the ALERT project was used to construct the continuous composite respectful maternity care score, which captures both respectful treatment and health system responsiveness.^35^ The score reflects women’s reported experiences across the six domains; absence of physical and verbal abuse, privacy and confidentiality, dignity and respect, communication and supportive care, social support and hospital environment, recognising that childbirth care is shaped by both interpersonal interactions and the care environment. Hospital environment and social support were retained as distinct domains based on the psychometric validation and their relevance to overall care experiences. Summary scores were calculated using simple summation and factor-weighted methods to assess the robustness of the scoring approach across study settings.

**Table 1:** Respectful maternity care items included in the composite score by education status and facilities

Question	Facility 1, N=253		Facility 2, N=249		Facility 3, N=251		Facility 4, N=251		Total, N=1004	
Physical and verbal abuse	Primary/no education	Secondary/ higher	Primary/no education	Secondary/ higher	Primary/no education	Secondary/ higher	Primary/no education	Secondary/ higher	Primary/no education	Secondary/ higher
Were you slapped or pinched by a provider? (yes)	2/178 (1.1%)	0/75 (0.0%)	2/157 (1.3%)	0/91 (0.0%)	0/194 (0.0%)	0/55 (0.0%)	3/186 (1.6%)	0/64 (0.0%)	7/715 (1.0%)	0/285 (0.0%)
Did a provider make any negative comments eg, about age/marital status/ethnicity/religion/HIV status? (yes)	1/178 (0.6%)	1/75 (1.3%)	2/156 (1.3%)	1/91 (1.1%)	0/195 (0.0%)	0/55 (0.0%)	1/187 (0.5%)	1/63 (1.6%)	4/716 (0.6%)	3/284 (1.1%)
Were you shouted at or told off because you did not bring items with you? (yes)	2/178 (1.1%)	1/75 (1.3%)	2/157 (1.3%)	2/91 (2.2%)	0/195 (0.0%)	2/55 (3.6%)	3/187 (1.6%)	1/63 (1.6%)	7/717 (1.0%)	6/284 (2.1%)
Did any of the providers or other staff suggest or ask you (or your family) for a bribe, informal payment or gift? (yes)	0/178 (0.0%)	0/75 (0.0%)	1/158 (0.6%)	1/90 (1.1%)	0/194 (0.0%)	0/56 (0.0%)	0/187 (0.0%)	0/64 (0.0%)	1/717 (0.1%)	1/285 (0.4%)
Maintained privacy and confidentiality										
Was your private or personal information shared without my consent? (yes)	2/178 (1.1%)	3/75 (4.0%)	2/158 (1.3%)	0/91 (0.0%)	6/195 (3.1%)	1/56 (1.8%)	1/187 (0.5%)	1/64 (1.6%)	11/718 (1.5%)	5/286 (1.7%)
Was your physical privacy violated eg, being uncovered or having people in the delivery room without my consent? (yes)	3/178 (1.7%)	3/75 (4.0%)	3/158 (1.9%)	1/91 (1.1%)	7/195 (3.6%)	1/56 (1.8%)	2/187 (1.1%)	3/64 (4.7%)	15/718 (2.1%)	8/286 (2.8%)
Maintained respect and dignity										
Did the providers at the hospital treat you with respect? (yes)	168/178 (94.4%)	71/75 (94.7%)	147/158 (93.0%)	84/91 (92.3%)	193/195 (99.0%)	53/56 (94.6%)	179/187 (95.7%)	61/64 (95.3%)	687/718 (95.7%)	269/286 (94.1%)
Did the providers at the hospital treat you in a friendly manner? (yes)	163/178 (91.6%)	70/75 (93.3%)	144/158 (91.1%)	83/91 (91.2%)	192/195 (98.5%)	53/56 (94.6%)	179/187 (95.7%)	63/64 (98.4%)	678/718 (94.4%)	269/286 (94.1%)
How would you describe the waiting time before you were admitted to the labour ward? (long)	41/178 (23.0%)	15/75 (20.0%)	32/158 (20.3%)	28/91 (30.8%)	27/195 (13.8%)	11/56 (19.6%)	24/187 (12.8%)	11/64 (17.2%)	124/718 (17.3%)	65/286 (22.7%)
Did you feel the providers at the hospital took the best care of you that they could? (yes)	171/178 (96.1%)	72/75 (96.0%)	147/158 (93.0%)	85/91 (93.4%)	192/195 (98.5%)	55/56 (98.2%)	183/187 (97.9%)	61/64 (95.3%)	693/718 (96.5%)	273/286 (95.5%)
Would you recommend a family member to give birth in the same hospital? (yes)	163/178 (91.6%)	68/75 (90.7%)	141/158 (89.2%)	83/91 (91.2%)	194/195 (99.5%)	52/56 (92.9%)	184/187 (98.4%)	61/64 (95.3%)	682/718 (95.0%)	264/286 (92.3%)
Were you shouted or screamed at by a provider or other member of staff? (yes)	14/178 (7.9%)	10/75 (13.3%)	6/157 (3.8%)	8/91 (8.8%)	8/195 (4.1%)	5/55 (9.1%)	12/187 (6.4%)	4/63 (6.3%)	40/717 (5.6%)	27/284 (9.5%)
Communication and supportive care										
Did the providers introduce themselves to you with their names when they first came to see you? (yes)	20/178 (11.2%)	5/75 (6.7%)	17/158 (10.8%)	21/91 (23.1%)	14/195 (7.2%)	6/56 (10.7%)	20/187 (10.7%)	14/64 (21.9%)	71/718 (9.9%)	46/286 (16.1%)
Did you feel the providers explained to you what had been done to you? (yes)	145/178 (81.5%)	64/75 (85.3%)	133/158 (84.2%)	80/91 (87.9%)	169/195 (86.7%)	50/56 (89.3%)	164/187 (87.7%)	59/64 (92.2%)	611/718 (85.1%)	253/286 (88.5%)
Did the provider ask you for permission before carrying out a vaginal examination? (yes)	155/178 (87.1%)	70/75 (93.3%)	143/158 (90.5%)	87/91 (95.6%)	178/195 (91.3%)	51/56 (91.1%)	166/187 (88.8%)	59/64 (92.2%)	642/718 (89.4%)	267/286 (93.4%)
Did the provider explain to you why they were carrying out examinations or procedures? (yes)	145/178 (81.5%)	64/75 (85.3%)	133/158 (84.2%)	80/91 (87.9%)	169/195 (86.7%)	50/56 (89.3%)	164/187 (87.7%)	59/64 (92.2%)	611/718 (85.1%)	253/286 (88.5%)
Did the provider explain to you why they were giving you any medicine? (yes)	98/121 (81.0%)	47/52 (90.4%)	95/110 (86.4%)	70/74 (94.6%)	98/115 (85.2%)	33/38 (86.8%)	101/122 (82.8%)	44/49 (89.8%)	392/468 (83.8%)	194/213 (91.1%)
Did you feel you could ask the provider at the hospital any questions you had? (yes)	147/178 (82.6%)	57/75 (76.0%)	125/158 (79.1%)	76/91 (83.5%)	158/195 (81.0%)	49/56 (87.5%)	151/187 (80.7%)	53/64 (82.8%)	581/718 (80.9%)	235/286 (82.2%)
Did the provider at the hospital talk to you about how you were feeling? (yes)	134/178 (75.3%)	55/75 (73.3%)	132/158 (83.5%)	75/91 (82.4%)	164/195 (84.1%)	48/56 (85.7%)	163/187 (87.2%)	57/64 (89.1%)	593/718 (82.6%)	235/286 (82.2%)
Did the providers at the hospital address your anxiety and fears	120/178 (67.4%)	47/75 (62.7%)	109/158 (69.0%)	57/91 (62.6%)	148/195 (75.9%)	42/56 (75.0%)	137/187 (73.3%)	54/64 (84.4%)	514/718 (71.6%)	200/286 (69.9%)
Were you encouraged to walk around during labour? (yes)	145/178 (81.5%)	55/75 (73.3%)	108/158 (68.4%)	73/91 (80.2%)	171/195 (87.7%)	51/56 (91.1%)	152/187 (81.3%)	48/64 (75.0%)	576/718 (80.2%)	227/286 (79.4%)
Were you encouraged to eat and drink during labour? (yes)	110/178 (61.8%)	45/75 (60.0%)	98/158 (62.0%)	64/91 (70.3%)	142/195 (72.8%)	43/56 (76.8%)	131/187 (70.1%)	48/64 (75.0%)	481/718 (67.0%)	200/286 (69.9%)
When you needed help, did you feel the providers at the hospital paid attention? (yes)	164/178 (92.1%)	67/75 (89.3%)	142/158 (89.9%)	82/91 (90.1%)	183/195 (93.8%)	55/56 (98.2%)	174/187 (93.0%)	63/64 (98.4%)	663/718 (92.3%)	267/286 (93.4%)
Social support										
Were you allowed to have someone you wanted (family/friend) to stay with you during labour and contractions (first stage of labour, the time before pushing)? (yes)	28/178 (15.7%)	11/75 (14.7%)	86/158 (54.4%)	48/91 (52.7%)	28/195 (14.4%)	10/56 (17.9%)	159/187 (85.0%)	49/64 (76.6%)	301/718 (41.9%)	118/286 (41.3%)
Were you allowed to have someone you wanted (family/friend) to stay with you during birth (second stage of labour, pushing)? (yes)	17/178 (9.6%)	8/75 (10.7%)	78/158 (49.4%)	43/91 (47.3%)	13/195 (6.7%)	4/56 (7.1%)	131/187 (70.1%)	36/64 (56.2%)	239/718 (33.3%)	91/286 (31.8%)

The primary outcome was overall respectful maternity care level derived from a continuous score that was subsequently dichotomised at the sample’s 25th percentile (84.5/100) to identify women reporting comparatively less respectful maternity care. Higher scores indicated more respectful and responsive care, such that women with scores at or below 84.5 were classified as reporting comparatively less respectful maternity care.

Questionnaire items used different response formats, including Likert-type, yes/no, categorical and 7-point recommendation scales. All items were recoded so that higher values reflected more respectful and responsive care, then normalised to a common 0–1 scale before subscale scores were calculated by dividing observed values by the maximum possible. Non-ordered responses, such as ‘not relevant’, ‘don’t know’ and ‘don’t want to say’, were excluded from scoring. Subscale scores were calculated from normalised items using the validated ALERT scoring approach, through either simple summation or factor-weighted scoring and rescaled to a 0–10 metric. The overall composite was expressed as a percentage of best possible care, with higher scores indicating better care experiences ensuring items with differing ranges contributed comparably. For regression analyses, the composite score was dichotomised at the 25th percentile, corresponding to 84.5 out of 100. Women scoring at or below this threshold were classified as reporting poorer respectful maternity care, representing the lowest quartile rather than a clinically validated cut-off.^35 38 39^

Descriptive analyses included individual score items, continuous and domain-specific respectful maternity care scores, selected care indicators and variation in reported care experiences across study settings. Maternal education was the main exposure, categorised as primary/no education versus secondary/higher. Additional demographic and care-related variables from the ALERT perinatal e-registry included maternal age, parity, mode of birth, employment status, facility and intervention period.^40^ Employment was grouped as paid, self-employed or unpaid; parity as primiparous, para 2, or ≥3; and mode of birth as spontaneous vaginal, caesarean or assisted. These categories were selected to support interpretability and adequate sample sizes. Intervention period was modelled as a contextual temporal covariate reflecting the ALERT stepped-wedge schedule, based on facility-specific crossover timing and delivery or interview date, as this analysis was not designed to estimate causal effects.

### Statistical analysis

All data were exported from REDCap to Stata V.18.0 for data cleaning and analysis. Descriptive statistics were used to summarise participants’ characteristics, including age, parity, education, employment, mode of birth and birth outcomes. Selected indicators of disrespectful care were also summarised to estimate their prevalence overall, by education level and by facility.

Logistic regression was used to examine the association between maternal education and reported less respectful maternity care. Both unadjusted and adjusted models were fitted. The adjusted model controlled for age and intervention as confounders. Age was identified from previous evidence and a directed acyclic graph, see acyclic graph in online supplemental file 2.^27 41^ The intervention was included as a fixed effect in the analysis model and was based on the stepped-wedge ALERT design and included simulation-based team training, digital audit and feedback, mentorship and facility-led quality improvement cycles.^28^ Results from the primary adjusted model are presented as ORs with 95% CIs. Statistical significance was set at p<0.05.

Four sensitivity analyses were conducted to assess the robustness of the primary findings and are presented alongside the primary model in table 2. First, because categorising the respectful maternity care score as a binary outcome may lose information, the association between education and the continuous respectful maternity care score was examined using linear regression and reported as a beta coefficient. Second, because the binary outcome was not rare, a modified Poisson regression with robust SEs was used to estimate prevalence ratios (PRs), which provide a more direct measure of relative association than ORs. Third, a narrower abuse-focused outcome was created to assess whether the association was driven by direct experiences of mistreatment. Women were classified as mistreated if they reported at least one abuse-related event, including physical or verbal abuse, non-consented care, neglect or violation of privacy. This outcome excluded broader experience-of-care measures, such as communication, supportive care, dignity and hospital environment, which were included in the main respectful maternity care composite. The narrower outcome was analysed using logistic regression. Fourth, the same abuse-focused outcome was analysed using modified Poisson regression with robust SEs to estimate a PR.

**Table 2:** Association between educational attainment and reported less respectful maternity care

Variable	Unadjusted OR	95% CI	P value	Adjusted OR	95% CI	P value
Educational attainment (Ref: primary/no education)						
Secondary/higher	1.54	1.06 to 2.24	0.02	1.48	1.02 to 2.15	0.04*

N=970 (unadjusted model), N=933 (adjusted model).

*Adjusted for age and intervention period.

Missing data were handled using complete-case analysis. Model fit was assessed using likelihood ratio tests and pseudo-R² statistics.

### Patient and public involvement

Community engagement activities were conducted prior to commencement of the broader ALERT study to inform stakeholders and strengthen acceptability. Patients and the public were not involved in study design, conduct or manuscript preparation. Postpartum women participated only as respondents after providing written consent. Findings will be shared in aggregate through feedback meetings with facilities, providers, district health teams and maternal health stakeholders in Malawi. Because survey data were anonymised, results cannot be returned to individual participants.

## Results

We approached a total of 1006 women for participation in the study. Of these, two women refused to provide consent and were excluded. Of the 1004 women included in the descriptive analysis, 970 had complete data for the unadjusted association between education and less respectful maternity care and 933 had complete data for all variables included in the adjusted model (see figure 1 online supplemental files figure). Regression analyses were therefore conducted using complete cases.

**Figure 1:**
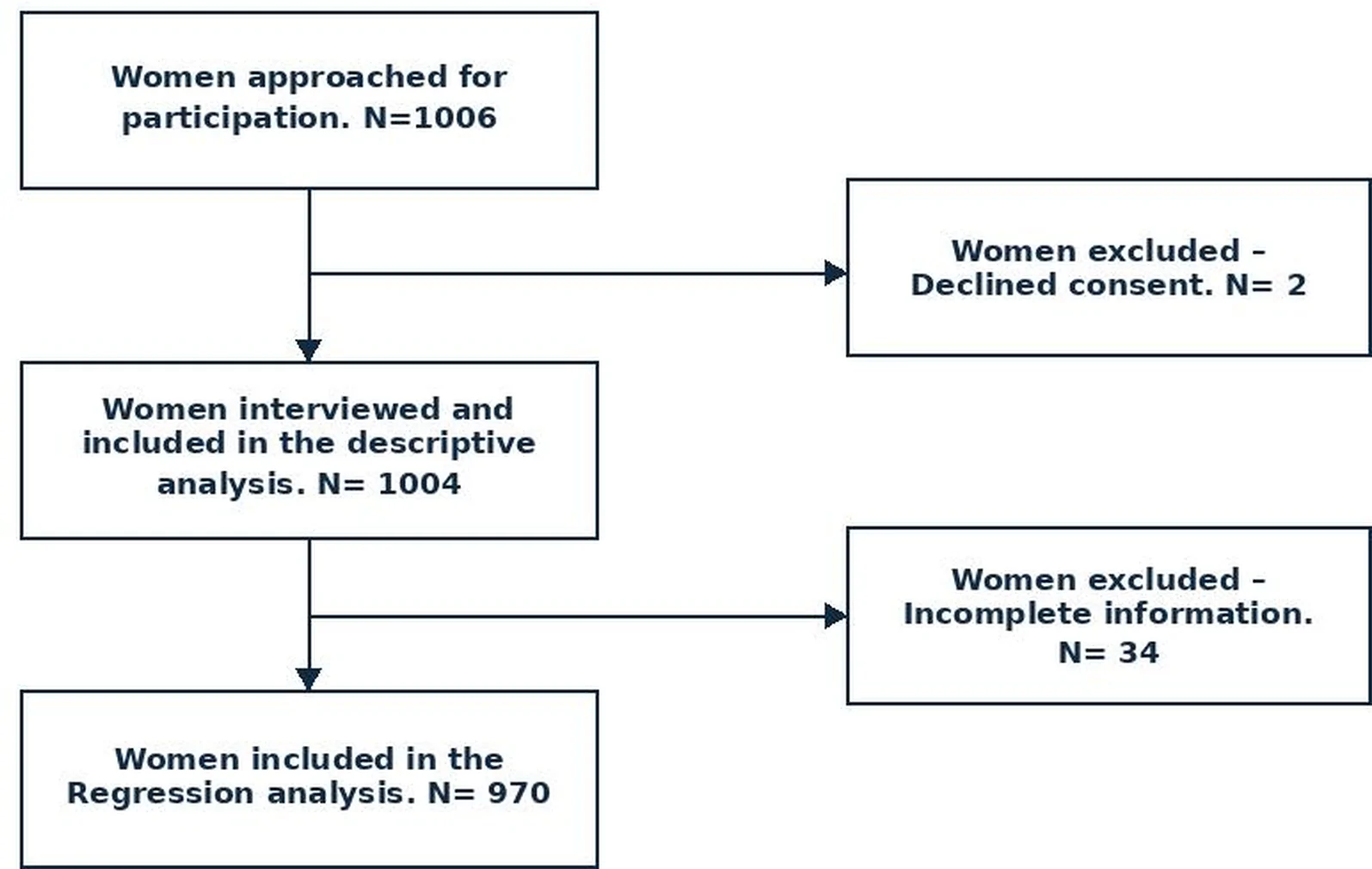
Participants flow diagram showing recruitment, exclusions and final analytical sample.

Table 3 summarises facility characteristics and the demographic and obstetrical profiles of the 1004 women included across four hospitals. Three facilities were public and one was faith-based, with annual delivery volumes ranging from about 2500–6500 births. Participants had a mean age of 24 years (SD 6.3) and a median parity of two. Most women (71.5%) had primary or no formal education, with Facility 2 showing the highest proportion of women with secondary or higher education.

**Table 3:** Characteristics of participants by facility (N=1004)

Variable	Facility 1 (N=253)	Facility 2 (N=249)	Facility 3 (N=251)	Facility 4 (N=251)	Total (N=1004)
Facility characteristics					
Type of facility	Public hospital	Public hospital	Public hospital	Faith-based hospital	
Mean annual births (approximately)	~5000–6000	~5500–6500	~4000–5000	~2500–3000	
Social demographics					
Age mean (SD)	24.7 (6.9)	24.5 (6.1)	23.3 (5.6)	23.8 (6.5)	24.1 (6.3)
Education					
Primary or no education	178 (70.4%)	158 (63.5%)	195 (77.7%)	187 (74.5%)	718 (71.5%)
Secondary or higher	75 (29.6%)	91 (36.5%)	56 (22.3%)	64 (25.5%)	286 (28.5%)
Parity					
Para 2	130 (51.4)	147 (59.0)	122 (48.6)	110 (43.8)	509 (50.7)
Para 3 and above	25 (9.9)	21 (8.4)	21 (8.4)	25 (10.0)	92 (9.2)
Employment type					
Paid employee	10 (4.0%)	10 (4.0%)	6 (2.4%)	10 (4.0%)	36 (3.6%)
Temporary/casual worker	20 (7.9%)	31 (12.5%)	33 (13.2%)	33 (13.2%)	117 (11.7%)
Self-employed	124 (49.0%)	97 (39.0%)	111 (44.2%)	105 (41.8%)	437 (43.5%)
Unpaid/unemployed	99 (39.1%)	111 (44.6%)	101 (40.2%)	103 (41.0%)	414 (41.2%)
Obstetric and birth outcomes					
Mode of birth					
Spontaneous vaginal	205 (83.3%)	217 (87.2%)	231 (92.0%)	171 (68.1%)	824 (82.7%)
Caesarean section	34 (13.8%)	28 (11.2%)	17 (6.8%)	73 (29.1%)	152 (15.3%)
Assisted birth	7 (2.9%)	4 (1.6%)	3 (1.2%)	7 (2.8%)	21 (2.1%)
Outcome of birth					
Alive	237 (96.3%)	244 (98.0%)	247 (98.4%)	243 (96.8%)	971 (97.4%)
Fresh stillbirth	6 (2.4%)	5 (2.0%)	2 (0.8%)	7 (2.8%)	20 (2.0%)
Macerated stillbirth	3 (1.2%)	0 (0.0%)	2 (0.8%)	1 (0.4%)	6 (0.6%)

Seven observations missing for mode and outcome of birth.

Education recoded to primary/none versus secondary/higher; employment to paid versus unpaid/unemployed.

Self-employment was the most common form of work (43.5%), while 41.2% of women were unemployed or unpaid. Only 3.6% reported formal paid employment, with 11.7% engaged in temporary or casual work. Most women (82.7%) had spontaneous vaginal births, while caesarean section rates varied by facility, peaking at 29.1% in Facility 4. Assisted births were uncommon (2.1%). The majority of deliveries resulted in live births (97.4%), with small proportions of fresh (2.0%) and macerated stillbirths (0.6%).

Table 1 presents women’s experiences of respectful maternity care across four hospitals, disaggregated by education. Direct abuse was rare, with fewer than 2% reporting slapping, pinching or informal payment requests. Verbal mistreatment was more frequent, particularly shouting, which affected 9.5% of women with secondary/higher education compared with 5.6% with primary/no education. Privacy violations were infrequent but slightly higher among more educated women. Overall, over 94% of respondents in both groups reported being treated with respect and friendliness, and most felt providers gave the best care possible. Yet gaps remained in long waiting times which were more common among women with secondary/higher education, provider self-introduction was low overall and social support varied widely by facility, with companion presence during birth reported by only one-third of women.

Table 4 presents the distribution of respectful maternity care scores by education group and facility. Of the 1004 women, 718 (71.5%) had primary/no education and 286 (28.5%) had secondary/higher education. Overall, the mean respectful maternity care score was 88.06 (SD 6.27) among women with primary/no education and 87.01 (SD 8.00) among those with secondary/higher education. The corresponding median (IQR) scores were 90.87 (4.0) and 90.08 (4.8), respectively. Facility-specific descriptive statistics are also presented in table 4.

**Table 4:** Descriptive patterns of the respectful maternity care score, by education group and facility

	Primary/no education	Secondary/higher education	All
Overall			
N (%)	718 (71.5)	286 (28.5)	1004 (100.0)
Mean (SD)	88.06 (6.27)	87.01 (8.00)	87.76 (6.82)
Median (IQR)	90.87 (87.70–91.67)	90.08 (86.90–91.67)	90.87 (86.90–91.67)
Facility 1			
N (%)	178 (70.4)	75 (29.6)	253 (100.0)
Mean (SD)	87.34 (6.46)	86.33 (8.17)	87.04 (7.01)
Median (IQR)	90.08 (86.90–91.67)	90.08 (85.32–91.67)	90.08 (86.11–91.67)
Facility 2			
N (%)	158 (63.5)	91 (36.5)	249 (100.0)
Mean (SD)	87.17 (7.65)	87.23 (8.02)	87.19 (7.77)
Median (IQR)	90.87 (86.90–90.87)	90.08 (87.70–90.87)	90.08 (86.90–90.87)
Facility 3			
N (%)	195 (77.7)	56 (22.3)	251 (100.0)
Mean (SD)	88.76 (4.86)	86.83 (8.58)	88.33 (5.93)
Median (IQR)	90.87 (88.49–91.67)	90.87 (87.30–91.67)	90.87 (88.29–91.67)
Facility 4			
N (%)	187 (74.5)	64 (25.5)	251 (100.0)
Mean (SD)	88.76 (5.99)	87.66 (7.35)	88.48 (6.36)
Median (IQR)	90.87 (88.69–91.67)	90.87 (86.71–91.67)	90.87 (88.49–91.67)

### Proportion of mistreatment items included in the score

Overall, across the included facilities, 16% of women were classified as experiencing less respectful maternity care according to the composite measure. Verbal abuse was the most reported item in all facilities (6.4–10.7%). Non-consented care or neglect was the second most frequent item ranging from (2.0% in facility 3–4.8% In Facility 2). Privacy violations were less common, while physical abuse and bribery were rare (see online supplemental file 4).

Table 2 presents the unadjusted and adjusted associations between women’s characteristics and the odds of reporting less respectful maternity care during childbirth. In the unadjusted analysis, women with secondary or higher education had higher odds of reporting less respectful maternity care than women with primary or no education (OR 1.54, 95% CI 1.06 to 2.24; p=0.02). This association remained statistically significant after adjustment for age and intervention period (adjusted OR 1.48, 95% CI 1.02 to 2.15; p=0.04).

Table 5 presents sensitivity analyses examining the association between secondary/higher maternal education and reported less respectful maternity care. In the continuous outcome model, women with secondary or higher education had lower respectful maternity care scores than women with primary or no education (β=−1.06, 95% CI −2.11 to −0.01; p=0.05), indicating an association in the same direction as the primary analysis. Similarly, the modified Poisson model using the binary composite outcome showed a higher prevalence of reported less respectful maternity care among women with secondary/higher education (PR=1.38, 95% CI 1.02 to 1.87; p=0.04). In contrast, neither of the two models using the narrower abuse-focused outcome comprising six mistreatment items: slapping, negative comments, shouting, mocking, privacy violations and physical privacy breaches, showed evidence of a statistically significant association with education: the logistic model gave an OR of 1.33 (95% CI 0.86 to 2.07; p=0.20), while the modified Poisson model gave a PR of 1.29 (95% CI 0.88 to 1.89; p=0.20).

**Table 5:** Sensitivity analyses for the association between secondary/higher maternal education and reported less respectful maternity care

Model	Estimate for secondary/higher education	95% CI	P value
Continuous respectful maternity care score	β=−1.06	−2.11 to −0.01	0.05
Modified Poisson model using binary composite outcome	PR=1.38	1.02 to 1.87	0.04
Narrow abuse-focused outcome, logistic model	OR=1.33	0.86 to 2.07	0.20
Narrow abuse-focused outcome, modified Poisson model	PR=1.29	0.88 to 1.89	0.20

Adjusted for age and intervention*.*

PR, prevalence ratio.

## Discussion

This study examined women’s reported respectful maternity care, including individual indicators of mistreatment during childbirth across four high-volume hospitals in Malawi, with a focus on educational differences using a validated respectful maternity care score. Overall, 16% were classified as reporting less respectful maternity care. Although direct physical abuse and informal payments were uncommon, gaps in communication, consent, emotional support, waiting times and companionship were evident, with shouting the most frequently reported form of verbal mistreatment. Women with secondary or higher education had higher odds of reporting less respectful maternity care. Sensitivity analyses indicated that this association was more apparent for the broader composite measure of care than for outcomes restricted to direct abuse.

The proportion classified as reporting less respectful maternity care cannot be directly compared with published prevalence estimates of mistreatment because the composite outcome and its lowest-quartile threshold capture a broader and differently defined construct.^4 42^ Nevertheless, individual indicators of verbal abuse, non-consented care and privacy violations were reported by a minority of women, while broader gaps were evident in communication, emotional support and social support, echoing findings from Malawi, Tanzania and Kenya.^4 6 25 19 43^ Variations likely reflect differences in measurement tools, interview timing, cultural norms and facility types.^4 44 45^ These results reinforce that experiences of maternity care are multidimensional, encompassing not only overt abuse but also deficits in communication, consent, emotional support and companionship. Early work by Bowser and Hill framed disrespect and abuse as a rights and quality-of-care issue, expanded by Freedman *et al* and Bohren *et al* into typologies of abuse and systemic constraints.^4 13 46^ WHO similarly defines respectful care as preserving dignity, privacy, informed choice and continuous support.^1 2 47^ Thus, low direct abuse does not imply consistently person-centred care.^2 4 24^

The finding that women with secondary or higher education had higher odds of reporting less respectful maternity care contrasts with evidence from several low- and middle-income settings, where women with lower levels of education have been found to be more vulnerable to mistreatment during childbirth. For example, the WHO multicountry study in Ghana, Guinea, Myanmar and Nigeria found that social disadvantage, including lower educational attainment, was associated with greater exposure to mistreatment, while systematic reviews have similarly reported higher risks among women with no formal education.^12 48^ In contrast, the association observed in our study was apparent when broader dimensions of respectful and responsive care were considered, rather than when attention was restricted to direct abuse.

One possible explanation is that education may influence women’s expectations of care and how they recognise and report shortcomings in communication, consent, dignity and support. Women with higher levels of education may have greater awareness of their rights and stronger expectations regarding participation in decisions and communication with providers. They may also feel more able to question aspects of care that they perceive as inadequate. Previous studies from Nepal, Ghana, Tanzania and Nigeria suggest that education and empowerment can influence how women recognise and evaluate disrespectful care, while evidence from other settings indicates that women with greater education may have lower tolerance for negative provider attitudes.^44 49 50^ Conversely, lower reporting among women with less education may reflect differences in recognition, expectations or willingness to report poor treatment rather than an absence of negative experiences.

Education may also shape interactions between women and providers. In hierarchical and resource-constrained maternity care settings, women who question or challenge decisions may encounter dismissive responses or tensions in their interactions with providers. Evidence from Kenya, Ethiopia and Tanzania suggests that education can increase women’s confidence and agency yet increase vulnerability to poor care.^51 52^ In Malawi, organisational norms and chronic resource constraints similarly reinforce acceptance of poor treatment as inevitable.^53^

Our sensitivity analyses support the interpretation that the association between secondary or higher education and less respectful maternity care was evident across broader measures of care experiences including communication, support, dignity and the care environment. Women with secondary or higher education had lower scores for respectful maternity care in the continuous model and were more likely to report less respectful maternity care in the modified Poisson model using the binary composite outcome. In contrast, when the outcome was restricted to a narrower set of direct abuse-related events, no statistically significant association with education was observed in either the logistic model or the modified Poisson model. Together, these findings suggest that educational differences were more apparent in broader assessments of respectful and responsive maternity care than in reports of overt abuse.

Therefore, our findings likely reflect differences in awareness, expectations and reporting of care experiences rather than differences in exposure to severe mistreatment. Women with higher levels of education may be better able to recognise aspects of care that do not meet expected standards and may therefore be more likely to report shortcomings in communication, respect and support during childbirth.^12 54 55^

This pattern also raises the possibility that differences in reporting and interpretation contributed to the observed association. Respectful maternity care measures include subjective assessments of communication, dignity, participation and emotional support and women with different educational backgrounds may interpret or evaluate these experiences differently. In psychometrics, such differences may be described as differential item functioning, whereby respondents with similar underlying experiences differ in their likelihood of endorsing items because of group characteristics.^56 57^ Education may influence expectations, comprehension, reporting thresholds and willingness to classify care as inadequate.^58^ Thus, associations between education and respectful care scores may reflect measurement non-equivalence. Literature on patient-reported outcomes stresses assessing invariance, as biased item functioning undermines validity.^59–62^ This is important because respectful care items often rely on women’s appraisal of interpersonal care. Direct events such as physical abuse or bribery may be less interpretive, whereas communication, dignity, consent and emotional support may vary by education-related expectations and reporting thresholds. However, measurement invariance and differential item functioning were not directly assessed in this study, and this explanation should therefore be regarded as a hypothesis rather than a demonstrated source of bias.

Taken together, the findings suggest that the association between education and less respectful maternity care should not be interpreted simply as evidence that more educated women experience greater exposure to mistreatment. Rather, it may reflect a combination of differences in care experiences, expectations, recognition and reporting of broader dimensions of maternity care. This distinction is important because respectful maternity care extends beyond the absence of overt abuse to include dignified, responsive, compassionate and woman-centred care.^24 34^

These findings highlight the importance of assessing respectful maternity care using broader measures that capture different dimensions of women’s care experiences. Measuring maternity care solely through direct abuse may overlook important deficits in communication, consent, emotional support, responsiveness and companionship. In this study, the association between education and reported less respectful maternity care was evident with the broader composite outcome but not with the narrower abuse-focused measure, suggesting that education-related differences may extend beyond experiences of direct abuse. The association was also more evident in the binary than the continuous analysis. This may reflect the different information captured by the two approaches: the binary outcome identified women in the lowest quartile of care scores, whereas the continuous outcome captured variation across the full score distribution.

Although the findings may indicate that educational differences are more apparent among women experiencing less respectful maternity care, this interpretation should be treated cautiously because dichotomising a continuous outcome can introduce a threshold effect and reduce information.^38^ Lower reporting of less respectful maternity care among women with less education should therefore not necessarily be interpreted as evidence of better care, as differences in awareness of rights, expectations or willingness to question or report providers may also influence responses. Consistent with respectful maternity care frameworks, efforts to improve equity should therefore extend beyond preventing direct abuse to strengthening communication, informed consent, emotional support, companion-friendly care, women’s awareness of their rights, community engagement, social accountability and safe mechanisms for providing feedback.^4 24 34 63 64^

### Strengths and limitations

This study had several methodological strengths. It used a large, multifacility sample of 1004 postpartum women across four hospitals, with a very low refusal rate. Structured interviews were conducted by trained research assistants using a psychometrically validated tool that integrated items from the Bohren mistreatment framework and the Person-Centred Maternity Care scale. The composite measure captured multiple domains of respectful maternity care, including communication, dignity, privacy, social support, hospital environment and absence of abuse. Systematic sampling adapted to daily discharge volumes, tablet-based REDCap data collection with real-time validation and close supervision within the ALERT platform strengthened data quality, consistency and completeness. Immediate postnatal interviews helped reduce recall bias, while use of a directed acyclic graph supported confounder selection.

Several limitations should be noted. The cross-sectional design limits causal interpretation of the association between education and reported less respectful maternity care. Self-reported outcomes may be affected by social desirability bias, especially because interviews were conducted within health facilities, and immediate postpartum surveys may underestimate mistreatment compared with later community follow-up.^7 34^ Reporting may also differ by education level, as more educated women may more readily recognise and report poor care. Although validated, the tool may not fully capture subtle or normalised forms of mistreatment, such as coercion or neglect embedded in routine practice.^65^ The composite outcome also includes interpersonal and structural domains, meaning some women may be classified as experiencing poorer respectful care due to system-level constraints rather than direct provider mistreatment. Findings should therefore be interpreted alongside individual mistreatment indicators. Finally, unmeasured facility-level factors, including workload, staffing and leadership, may have influenced both experiences and reporting.

### Implications for practice and policy

These findings highlight the need for sustained investment in respectful maternity care in Malawi. The association between education and reported less respectful maternity care suggests that education may shape women’s ability to recognise, interpret and report less respectful maternity care, consistent with evidence that more educated women may be better positioned to identify and challenge poor care.^4 34^ Rights-based antenatal education, particularly for women with limited formal schooling, may strengthen awareness of acceptable standards of care and support more confident engagement with providers.^13^

Facility differences and lower reporting during the post-intervention period suggest that targeted investments in maternity care may improve women’s experiences. However, the intervention-period finding should be interpreted as a temporal association rather than evidence of ALERT impact. The ALERT approach, which included provider training, mentorship, monitoring and facility-led improvement activities, aligns with evidence that structured interventions can improve provider-patient interactions and reduce disrespectful practices.^66 67^

Improving respectful care also requires structural reforms beyond individual-level training. Chronic staffing shortages, poor working conditions, weak governance and inadequate infrastructure continue to undermine care quality.^1 65 68^ Recent initiatives in Malawi, such as delivery cubicles with curtains and bathrooms, demonstrate responsiveness to women’s need for privacy and dignity.^69^ Sustained progress will require coordinated action across individual, facility and health-system levels, aligned with global and national respectful maternity care frameworks.^2 70^

## Conclusion

Suboptimal respectful maternity care remains a persistent challenge within Malawi’s maternity care system, with women continuing to report experiences ranging from verbal abuse to violations of privacy and gaps in consent and participation in care despite ongoing policy commitments to respectful maternity care. This study applied a validated multidomain respectful maternity care index across four ALERT-enrolled hospitals, capturing women’s experiences of dignity, communication, privacy, social support, hospital environment and freedom from abuse. By assessing care as a multidimensional construct, the index revealed variation by education, suggesting that recognition, expectations, care-seeking patterns and organisational culture shape both experiences and reporting. The analysis showed that women with higher levels of education had higher odds of reporting poorer respectful maternity care, underscoring the role of awareness and expectations in hierarchical, resource-constrained systems. These findings provide evidence of the feasibility and value of composite measures in implementation research. Improving respectful maternity care requires systemic investments in workforce training, infrastructure and governance to ensure that every woman receives dignified, responsive and equitable care, a clinical obligation and fundamental human right.

## Supplementary material

10.1136/bmjopen-2026-118574 online supplemental file 1

10.1136/bmjopen-2026-118574 online supplemental file 2

10.1136/bmjopen-2026-118574 online supplemental file 3

10.1136/bmjopen-2026-118574 online supplemental file 4

10.1136/bmjopen-2026-118574 online supplemental file 5

## Data Availability

Data are available upon reasonable request.
