# Supplementary material for: Respectful care during childbirth and educational inequities: a cross-sectional survey using a multidomain index across four hospitals in Malawi
Source: BMJ Open. 2026 Oct 21;16(10):e118574. doi: 10.1136/bmjopen-2026-118574 (PMC13691797; doi:10.1136/bmjopen-2026-118574)
Supplement: Supplementary file 1 [file bmjopen-16-10-s001.pdf]

**Supplemental file 1: Study tool**

## Mistreatment Questions Tool

| Subscore | Item | Scale | Taken from | Changes to original tool |
| --- | --- | --- | --- | --- |
| Maintained respect & dignity | Did the providers at the hospital treat you with respect? | 0=No, never; 1=Yes, a few times; 2=Yes, most of the time; 3=Yes, all of the time | Afulani et al. and Bohren et al. | Bohren used agree–disagree scale; Afulani's scale followed |
| | Did the providers at the hospital treat you in a friendly manner? | 0=No, never; 1=Yes, a few times; 2=Yes, most of the time; 3=Yes, all of the time | Afulani et al. | Used "providers" instead of doctors, nurses, or staff |
| | How would you describe the waiting time before you were admitted to the labour ward? | 0=Very short; 1=A little long; 2=Somewhat long; 3=Very long | Afulani et al. | Reformulated from original question wording (How did you feel about the amount of time you waited? Would you say it was...) |
| | Were you shouted or screamed at by a provider or staff? | 0=No; 1=Yes; 2=Don't want to say; 3=Don't know | Bohren et al. | None |
| | Were you mocked by a provider or staff? | 0=No; 1=Yes; 2=Don't want to say; 3=Don't know | Bohren et al. | None |
| | Did you feel the providers took the best care of you they could? | 0=No, never; 1=Yes, a few times; 2=Yes, most of the time; 3=Yes, all of the time | Afulani et al. | None |

| | | | | |
|---|---|---|---|---|
| | Would you recommend a family member to give birth in the same hospital? | 1=Very strongly recommend … 7=Very strongly do not recommend | Bohren et al. | Modified scale; Bohren also asked about overall satisfaction |
| Maintained privacy & confidentiality | Was your personal information shared without consent? | 0=No, never … 3=Yes, all of the time | Bohren et al. | Reformulated wording from original (Did a staff member discuss your private information about your health in a way that others could hear?) |
| | Was your physical privacy violated (e.g., uncovered or extra people in the room)? | 0=No, never … 3=Yes, all of the time | Bohren et al. | Negative formulation vs. original; different scale (feel that my privacy was respected during examinations and treatments |
| Lack of physical/verbal abuse | Were you slapped or pinched by a provider? | 0=No; 1=Yes; 2=Don't want to say; 3=Don't know | Bohren et al. | Two questions combined (pinched and slapped) |
| | Did a provider make negative comments (e.g., age, marital status, religion, HIV)? | 0=No; 1=Yes; 2=Don't want to say; 3=Don't know | Bohren et al. | Options combined compared to original options age, marital status, ethnicity, religion and HIV compared to Bohren |
| | Were you shouted at or told off for not bringing items? | 0=No; 1=Yes; 2=Don't want to say; 3=Don't know | Neither | Both tools have a question on shouting but not particularly for lack of items |

| | | | |
|---|---|---|---|
| Did providers or staff ask for a bribe, informal payment, or gift? | 0=No; 1=Yes; 2=Don't want to say; 3=Don't know | Bohren et al. | None |
