# Supplementary material for: Respectful care during childbirth and educational inequities: a cross-sectional survey using a multidomain index across four hospitals in Malawi
Source: BMJ Open. 2026 Oct 21;16(10):e118574. doi: 10.1136/bmjopen-2026-118574 (PMC13691797; doi:10.1136/bmjopen-2026-118574)
Supplement: Supplementary file 2 [file bmjopen-2026-118574.reviewer_comments.pdf]

PEER REVIEW HISTORY

BMJ Open publishes all reviews undertaken for accepted manuscripts. Reviewers are asked to complete a checklist review form and are provided with free text boxes to elaborate on their assessment. These free text comments are reproduced below.

ARTICLE DETAILS

Title (Provisional)

Respectful Care During Childbirth and Educational Inequities: A Cross-sectional Survey Using a Multi Domain Index Across Four Hospitals in Malawi.

Authors

KANDEYA, BIANCA; Hanson, Claudia; El Halabi, Soha; Annerstedt, Kristi Sidney; Stones, William; Chipeta, Effie; Sun, Sunjuri

---

VERSION 1 - REVIEW

Reviewer 1

Name Mehrtash, Hedieh

Affiliation World Health Organization, Department of Sexual and Reproductive Health and Research, including UNDP/UNFPA/UNICEF/WHO/World Bank Special Programme of Research, Development and Research Training in Human Reproduction (HRP)

Date 02-Apr-2026

COI None

This manuscript addresses an important question in the field of respectful maternity care. The study's core strengths are notable: a large, multi-facility, prospectively collected sample embedded within a well-designed implementation research platform represents a relatively rare methodological asset in the sub-Saharan African maternity care literature, and the research question itself has direct relevance for policy and practice.

ABSTRACT

The abstract contains one inconsistency that should be corrected before publication. The primary outcome is described as "a binary mistreatment measure derived from a factor-weighted score capturing any abuse, neglect, non-consented care, discrimination, or major privacy breach," but this does not accurately

reflect the full six-domain composite described in the methods section, which additionally includes hospital environment and social support. The abstract should be revised to accurately represent how the outcome was constructed, as the current wording implies a narrower, abuse-focused measure that may be more defensible than what was used.

Additionally, the conclusion sentence in the abstract in which the education finding "reveals inequities in awareness" presents only one of two competing interpretations discussed in the paper. The alternative explanation, that more educated women may provoke provider hostility through assertive behavior, is also discussed in the body of the paper and should at minimum be acknowledged in the abstract conclusion, or the framing should be qualified with appropriate uncertainty.

METHODS

1. Primary Outcome Definition

The binary mistreatment outcome is derived by dichotomizing a continuous composite score at the 25th percentile. Several clarifications are needed:

The composite aggregates six domains including hospital environment and social support, which extend well beyond traditional definitions of mistreatment and validated measures that use domain-specific outcomes. This is a major methodological risk of the study to potentially misclassifying women based on structural deficiencies (e.g., long wait times, absent birth companion) vs than interpersonal abuse. The authors should strongly justify why these broader domains belong in a single mistreatment outcome, or present sensitivity analyses using a narrower abuse-focused measure.

The 25th percentile threshold is sample-dependent and not clinically interpretable. The authors should report what score values correspond to this cut-point and what they mean substantively. A sensitivity analysis using the continuous score would considerably strengthen the paper.

2. Questionnaire Validation

Several aspects of the validated instrument require further clarification: The psychometric validation is attributed to Morris et al. (2025, reference 35), which appears to be a paper produced by overlapping members of the same ALERT research team using data from the same platform but the questions in the supplement and methods section are from Bohren and Afulani et al papers. The authors should clarify whether the validation was conducted on an independent sample or on the same dataset used here, as the latter would constitute a form of circular validation that weakens the psychometric justification for the composite score.

Supplemental File 2 reveals considerable heterogeneity in response scales across items where some use 4-point Likert scales, others binary yes/no responses, and the recommendation item uses a 7-point scale. The authors do not explain how these were harmonized into a single composite score. Items with different response ranges will contribute unequally to a simple summation approach, and this should be addressed explicitly.

The tool was translated into Chichewa by the research team, but no detail is provided on the translation protocol. Whether back-translation, cognitive interviewing, or community piloting was conducted is not described. For a psychometric instrument used as the primary outcome, this is a notable gap and should be addressed.

3. Data Collection

Lines 135–141: The total number of discrete data collection rounds across the 27-month period is never stated. Given the stepped-wedge design with six-month intervals, this should be specified so readers can understand the temporal structure of the data.

Lines 142–143: Beyond stating the overall window (December 2021–March 2024), there is no information about when each facility crossed over from control to intervention which is the most critical temporal detail in a stepped-wedge design. The crossover sequence and timing for each facility should be provided, ideally in a supplementary timeline table.

Lines 146–148: It is unclear whether four-week collection periods occurred simultaneously across facilities or were staggered with the crossover schedule. If collection periods straddled a facility's crossover point, individual women could be misclassified as pre- or post-intervention introducing misclassification in a covariate that is statistically significant in the adjusted model (lines 288–290).

4. Sample Size and Power

Lines 143–146: The power calculation parameters (75% improvement from 4% baseline, ICC 0.1, 77% power) were derived for the ALERT trial's primary perinatal outcome, not for the mistreatment outcome examined here. Given that the adjusted odds ratio of 1.48 (95% CI 1.01–2.18) sits close to the boundary of significance, the authors should clarify whether the study was adequately powered to detect the education–mistreatment association.

The implied four to five collection rounds per facility over 27 months are never explicitly stated. The actual number of completed rounds per facility should be reported.

Three sampling fractions are described by discharge volume, but it is not stated whether volumes were stable across the 27-month period. If higher volumes coincided with particular intervention phases, the sampling fraction could vary systematically between pre- and post-intervention periods, introducing a selection bias not acknowledged in the analysis.

4. Analytic approach

The stepped-wedge structure introduces non-random temporal variation as hospitals transition to the intervention at different times, and women's experiences are not independent across time and facility. Furthermore, given the repeated measures across time , if understood correctly, standard logistic regression does not account for this clustering or the time-by-cluster interaction inherent to the design. The authors should clarify whether mixed-effects models or GEE approaches were considered and why complete-case logistic regression was deemed sufficient. The intervention period is treated as a simple binary covariate,

which is a considerable oversimplification of the stepped-wedge exposure structure. The authors should acknowledge this as a limitation and discuss the potential for residual confounding by time.

RESULTS

1. Descriptive Findings by Education: Table 2 presents raw frequencies for individual mistreatment items across facilities, but does not stratify by education which is the study's primary exposure. Adding education-stratified frequencies for the key items (shouting, privacy violations, non-consented care) would considerably strengthen the descriptive results and allow readers to assess whether the composite outcome direction is consistent across individual indicators.

2. Duplicate Reference: References 12 and 14 appear to be identical (Bohren et al., The Lancet, 2019). This should be corrected, as it affects the credibility of the citations supporting key results claims.

3. Flowchart Rendering: The participant flowchart appears to be rendered as plain text rather than as a figure, suggesting a formatting issue in the submitted proof. This should be corrected before publication.

4. Abstract–Methods Inconsistency: the abstract describes the primary outcome as "a binary mistreatment measure derived from a factor-weighted score capturing any abuse, neglect, non-consented care, discrimination, or major privacy breach," but this does not accurately reflect the full six-domain composite described in the methods section. The abstract should be revised to accurately represent how the outcome was constructed.

DISCUSSION

1. Interpretation of the Education–Mistreatment Association

This is the paper's central contribution and requires more precise engagement. The authors oscillate between two competing explanations that education increases awareness and recognition of mistreatment, and that education may provoke provider hostility through perceived non-compliance without attempting to distinguish between them empirically. Even a brief examination of whether the education effect differs across mistreatment subtypes (e.g., stronger for communication domains than for physical abuse items) would add meaningful analytical depth and help readers evaluate which mechanism is more plausible.

The finding that lower reporting among less-educated women likely reflects under-recognition rather than lower exposure is important and nuanced, but needs to be more prominent in both the abstract and conclusion. As currently written, the framing risks being read as suggesting more educated women face greater risk of mistreatment, which is almost certainly not the intended interpretation and could be misleading to non-specialist readers.

The discussion would also benefit from engaging with the measurement literature on differential item functioning (DIF) by education level. If more educated women systematically interpret and respond to identical care behaviors differently, this is a psychometric issue that cannot be resolved through narrative discussion alone and should be acknowledged explicitly as a core limitation.

2. Facility-Level Comparison

The faith-based facility (Facility 4) differs from the three government hospitals as it charges fees for service, serves a potentially different socioeconomic population, and operates under distinct governance and staffing norms. While the adjusted model controls for employment status as a proxy for socioeconomic position, it does not capture wealth, out-of-pocket expenditure, or care-seeking selectivity. Given that Facility 4 also has the lowest delivery volume and the highest caesarean section rate (29.1%), unmeasured case-mix differences may explain a portion of the observed facility effect. This should be acknowledged more explicitly as a limitation rather than treated as a straightforward cross-sector comparison.

Major revision is recommended, with the expectation that the analytic approach and outcome operationalization are substantially revised. Should the revised manuscript fail to adequately address the primary outcome construction and stepped-wedge analytic concerns, the manuscript's central finding cannot be considered sufficiently supported.

**Reviewer**	**2**
**Name**	**Malhotra, Nisha**
**Affiliation**	**The University of British Columbia**
**Date**	**28-Apr-2026**
**COI**	**None**

Overall Recommendation – Major Revisions on the objective and its contribution.

This paper makes a genuinely valuable methodological contribution that deserves to be foregrounded more clearly than the current framing allows. Its most important achievement is not the prevalence estimate per se, but the operationalization and application of a composite, multi-domain index of respectful maternity care.

The study goes well beyond narrow measures of mistreatment to operationalize a composite, multi-domain index of respectful maternity care. The index integrates dignity, autonomy, communication, privacy, social support, and hospital environment into a single psychometrically validated construct.

The composite index measures what Respectful maternity care looks like.

What this paper does well

This is conceptually richer than binary abuse measures and reflects best practice in patient-reported experience measurement. The instrument, validated within the ALERT platform (Morris et al., 2025, BMC Pregnancy & Childbirth), yields factor-weighted and summative scores that enable consistent comparison of care quality across facilities and over time. This is precisely the kind of standardized performance index that health systems

and facility managers need: one that can be administered in any hospital setting, scored consistently, and used to benchmark care against a meaningful threshold across countries and contexts.

The authors should consider reframing the paper around this contribution explicitly, positioning it as the development and deployment of a validated respectful maternity care performance tool, rather than primarily as a prevalence study.

The data collection is equally commendable. Structured interviews with 1,004 women across four facilities, a refusal rate of just 0.2%, tablet-based REDCap with real-time validation, and a systematically randomized sampling approach calibrated to facility discharge volumes: these represent a high standard of implementation research fieldwork that the authors should not undersell.

A necessary reframing of scope

However, the current framing invites interpretations that the study design cannot support, and revision on three points is essential.

First, this is not a study of the prevalence of mistreatment in Malawi's maternity care system. All four facilities were participants in the ALERT programme — an active quality-improvement intervention designed specifically to reduce disrespectful practices, with intensive supervision, provider training, and ongoing monitoring. Such facilities are not representative of routine hospital care. The 16% prevalence estimate characterizes care in a supported, actively observed set of hospitals, and cannot be generalized to Malawi's Central Region or nationally. Generalisability claims in the abstract, the Strengths section, and the Discussion should be revised accordingly.

Second, the paper does not evaluate the ALERT intervention. A credible impact evaluation requires baseline data from the same facilities or a comparable control group, neither of which is available here. The pre/post binary variable captures a temporal association within a stepped-wedge design — useful as a descriptive signal, but not a causal estimate. Language implying the programme reduced mistreatment should be softened to language describing an association consistent with a programme effect.

Third, and consequently, the intervention context shapes the null results. In facilities already enrolled in a quality-improvement programme — where care is actively monitored and providers are trained — disparities by employment, age, parity, and other sociodemographic characteristics are likely to be attenuated relative to what would be observed in routine care settings. The absence of significant associations for these variables may reflect the homogenizing effect of the intervention environment rather than a true absence of such gradients in Malawian maternity care. This caveat is currently absent from the Discussion and should be addressed directly.

In sum, this paper's strongest and most durable contribution is its measurement framework and its demonstration that a multi-domain respectful maternity care index can be applied consistently across diverse hospital settings, measured and analyzed.

The authors are encouraged to reframe the contribution, revise the scope and generalisability claims. None of this requires new data; it requires more precise language about what this sample can and cannot tell us.

Methods/Statistics

L.200–201 Logistic regression is used with a 16% outcome prevalence. At this prevalence level, odds ratios can overestimate risk ratios by approximately 15–20% (Zou, 2004; McNutt et al., 2003). The authors may wish to consider a modified Poisson regression with robust variance estimation as a sensitivity analysis, which would yield prevalence ratios that are directly interpretable as relative risks. If logistic regression is retained as the primary model, a brief sentence acknowledging the direction of overestimation would be appropriate.

Framing/Interpretation

L.317–329 The finding that more educated women report higher odds of mistreatment is interpreted as reflecting greater recognition and reporting capacity rather than greater exposure. This is a plausible and well-supported reading, consistent with literature cited. However, the cross-sectional design cannot rule out that educated women receive worse care (e.g., provider perception of assertive patients in hierarchical settings — the 'double-edged sword' point raised at L.330–338 is relevant here). The conclusion should be softened to acknowledge that the observed OR could reflect any combination of differential exposure, recognition, and reporting, and that the design cannot disentangle these pathways.

L.96–102 / L.293–305 The study is nested within the ALERT implementation research programme, a quality-improvement intervention specifically designed to strengthen intrapartum care and reduce disrespectful practices (L.96–102). This is important contextual information that may warrant more prominent discussion when interpreting the prevalence estimates and comparing them with figures from non-intervention settings. Facilities enrolled in an active implementation research programme are likely to differ systematically from the general population of Malawian hospitals

L.364–376: The Strengths section states that the large, multisite sample "enhances generalisability and enables examination of facility-level variation." The generalisability claim warrants more careful phrasing. All four facilities were participants in the ALERT implementation research programme; a purposively selected set engaged in active quality improvement. Purposive selection of intervention-enrolled facilities does not support broader generalization to Malawian hospitals not participating in ALERT, nor to the wider Central Region population.

The authors may wish to revise this sentence to separate these two distinct contributions — for example: "The multisite design enables rigorous examination of facility-level variation in care experiences within an implementation research context; findings should be interpreted with reference to the programme-enrolled nature of the sample rather than as population-representative estimates for Malawi."

This revision preserves the design's genuine strength while accurately characterizing its scope. — is well supported and a genuine contribution to the paper. However, the generalisability claims warrant more careful phrasing. All four facilities were participants in the ALERT implementation research programme; a purposively selected set engaged in active quality improvement. Purposive selection of intervention-enrolled facilities does not support broader generalisation to Malawian hospitals not participating in ALERT, nor to the wider Central Region population. The authors may wish to revise this sentence to separate these two distinct contributions — for example: “The multisite design enables rigorous examination of facility-level variation in care experiences within an implementation research context; findings should be interpreted with reference to the programme-enrolled nature of the sample rather than as population-representative estimates for Malawi.” This revision preserves the genuine strength of the design while accurately characterizing its scope.

VERSION 1 - AUTHOR RESPONSE

**Reviewer 1 - overall assessment:** The manuscript addresses an important question in respectful maternity care. The large multi-facility prospectively collected sample embedded within an implementation research platform is a notable strength and has direct relevance for policy and practice.	Thank you for recognising the importance of the research question and the strengths of our study design.
**Reviewer 1 - Abstract, outcome definition:** The abstract describes the primary outcome as a binary mistreatment measure capturing abuse, neglect, non-consented care, discrimination, or major privacy breach. This does not reflect the full six-domain composite, which also includes hospital environment and social support.	Thank you for this important clarification. We have revised the abstract to more accurately describe the primary outcome. It now specifies that reported mistreatment is derived from a binary composite respectful maternity care score encompassing all six domains: absence of physical and verbal abuse, privacy and confidentiality, respect and dignity, communication and supportive care, social support, and hospital environment. This change on lines 21-27 aligns the abstract with the outcome definition presented in the Methods section.
**Reviewer 1 - Abstract, education interpretation:** The abstract conclusion states that the education finding 'reveals inequities in awareness', but this presents only one interpretation. The alternative explanation that more educated women may experience provider hostility through perceived assertiveness should also be acknowledged or the framing qualified.	Thank you for this important comment. We have revised the abstract conclusion to avoid presenting awareness as the only explanation for the association between higher education and reported mistreatment. The revised wording now acknowledges that higher reporting among more educated women may reflect differences in exposure, recognition, expectations, willingness to report, or provider responses to perceived assertiveness. Changes have been made on lines 35-39. This brings the abstract in line with the more nuanced interpretation presented in the Discussion.

Reviewer comment	Response / action taken

**Reviewer 1 - Methods, primary outcome construction:** The composite aggregates six domains, including hospital environment and social support, which extend beyond traditional mistreatment definitions and may misclassify women based on structural deficiencies rather than interpersonal abuse. The authors should justify this choice or present sensitivity analyses using a narrower abuse-focused measure.	Thank you for this important comment. We agree that the six-domain composite reflects a broader construct than interpersonal mistreatment alone. We have revised the manuscript to clarify that the primary outcome is a **respectfulness score**, representing poorer reported respectful maternity care derived from a validated composite, rather than a narrow measure of abuse. The composite is retained because the ALERT validation identified six domains of women's childbirth care experiences, communication and supportive care, hospital environment, respect and dignity, social support, privacy and confidentiality, and absence of physical/verbal abuse, which together capture both interpersonal and health system dimensions. The changes have been made on lines 164-195. Sensitivity analyses using a narrower abuse-focused measure has been presented on lines 280-296. To address concerns about misclassification, we have strengthened the Methods to justify inclusion of hospital environment and social support, clarified that individual mistreatment items are reported separately as separate domains, and added this issue to the limitations. We also revised the wording throughout to refer to "poorer respectful maternity care" or "reported mistreatment based on a composite respectful care score," where appropriate.
**Reviewer 1 - Methods, 25th percentile threshold:** The 25th percentile threshold is sample-dependent and not clinically interpretable. The authors should report the score value corresponding to the cut-point and explain what it means substantively. A sensitivity analysis using the continuous score would strengthen the paper.	Thank you for this helpful comment. We have revised the Methods section to clarify the substantive meaning of the 25th percentile cut-off and to report the corresponding score value. The composite respectful maternity care score was calculated as a percentage of best practice, where 100 represents the optimal reported care experience and lower scores indicate poorer reported respectful maternity care, consistent with the scoring approach described in the ALERT validation paper. We now state that the 25th percentile corresponded to a score of 84.5 out of 100, meaning that women scoring at or below this value were classified as having reported poorer respectful maternity care relative to other women in the study sample. We agree that this threshold is sample-dependent and not a clinical cut-off, and we have made this limitation explicit on lines 181-184. The manuscript already explains that the continuous score was dichotomised at the 25th percentile because no validated clinical threshold exists for defining high mistreatment using composite experience-of-care measures. In response to the reviewer's suggestion, we have also added a sensitivity analysis using the continuous composite respectful maternity care score In Table 4. This allows the education–mistreatment association to be assessed without loss of information from dichotomising the score. The results of this sensitivity analysis have been added to the Statistical Analysis and Results sections and are interpreted alongside the binary outcome.
**Reviewer 1 - Methods, validation sample:** The psychometric validation is attributed to Morris et al. (2025), produced by overlapping ALERT team members using the ALERT platform. The authors should clarify whether validation was conducted on an independent sample or the same dataset, as the latter would weaken the psychometric justification.	Thank you for raising this important point. The psychometric validation reported in Morris et al. (2025) was conducted using ALERT data collected from 16 hospitals across Benin, Malawi, Tanzania, and Uganda. That validation study used a dataset of 2,433 women, randomly split (stratified by country) into two subsamples for exploratory and confirmatory factor analyses, thereby strengthening the psychometric justification through internal cross-validation despite being drawn from the same overall dataset.<br>By contrast, the present analysis uses additional data collected later, during the stepped-wedge ALERT trial, and is restricted to four facilities in Malawi. Our focus is on applying the selected items from the validated questionnaire within this national context to explore respectful maternity care in greater depth. We have clarified this distinction in the Methods section to avoid ambiguity and to highlight that while the validation was multi-country and earlier in time, the current study is Malawi-specific and temporally distinct.
**Reviewer 1 - Methods, heterogeneous item response scales:** Supplemental File 2 shows different response scales across items, including 4-point Likert, binary yes/no, and a 7-point recommendation item. The authors should explain how these were harmonised into a single composite score because different ranges may contribute unequally to summation.	Thank you for this important comment. We agree that the original manuscript did not provide sufficient detail on how items with different response scales were harmonised prior to constructing the composite score. We have revised the Methods section to clarify this process. Specifically, items were first recoded so that higher values consistently represented more respectful care and then normalised to a common 0–1 scale before calculating subscale and composite scores. This ensured comparability across binary, Likert-type, and 7-point response items and avoided undue weighting of items with broader response ranges. We have further clarified that subscale scores were derived from these normalised items and rescaled to a 0–10 metric, while the overall composite was expressed as a percentage of best possible care. These revisions align the manuscript with the validated ALERT scoring approach and make the harmonisation procedure more transparent. Changes have been made on lines 173-181.
**Reviewer 1 - Methods, translation protocol:** The tool was translated into Chichewa by the research team, but no detail is provided on the translation protocol, including back-translation, cognitive interviewing, or piloting.	Thank you for this comment; we have revised the Methods section to describe the translation procedures in greater detail, including forward translation into Chichewa by bilingual research team members with nursing and public health expertise, review by the local study team, pilot testing during training, minor wording adjustments, and ongoing supervision during data collection (lines 119–123). Although formal back translation was not conducted, these steps helped ensure conceptual accuracy, cultural relevance, consistent administration, and preservation of the intended meaning of the questionnaire items.
**Reviewer 1 - Data collection rounds:** Lines 135-141: The total number of discrete data collection rounds across the 27-month period is not stated. Given the stepped-wedge design with six-month intervals, this should be specified.	Thank you for this comment. We have revised the Methods section to specify the number of data collection rounds. The changes were made on lines 139-140 to show that the questionnaire was administered in **five discrete data collection rounds.**

Reviewer comment	Response / action taken
**Reviewer 1 - Crossover timing:** Lines 142-143: The manuscript gives the overall data collection window but not when each facility crossed from control to intervention. The crossover sequence and timing for each facility should be provided, ideally in a supplementary timeline table.	Thank you for highlighting the need for greater transparency regarding the stepped-wedge implementation schedule. We have revised the Methods section and added Supplementary File 5, which shows the facility-specific crossover sequence and timing. The table indicates when each hospital transitioned from control to intervention and how the five data collection rounds aligned with the ALERT rollout. This clarifies how observations were classified as pre- or post-intervention and reduces ambiguity around the timing of data collection changes have been made on lines 137-146.
**Reviewer 1 - Collection periods and misclassification:** Lines 146-148: It is unclear whether four-week collection periods occurred simultaneously across facilities or were staggered with the crossover schedule. If periods straddled crossover points, individual women could be misclassified as pre- or post-intervention.	Thank you for this important comment. We have revised the Methods section to clarify the organisation of data collection in relation to the ALERT stepped-wedge schedule and how intervention status was assigned. Data collection was conducted in discrete six-monthly rounds across the four hospitals, with four-week recruitment periods implemented according to the ALERT study timeline. This information has been provided on lines 137-146, along with a note on potential residual temporal confounding related to the step-wedged design
**Reviewer 1 - Sample size and power:** Lines 143-146: The power calculation was derived for the ALERT trial's primary perinatal outcome, not for the mistreatment outcome. Given the education adjusted OR of 1.48 (95% CI 1.01-2.18), the authors should clarify whether the study was adequately powered to detect the education-mistreatment association.	We agree. We revised the sample size section to clarify that the original power calculation was for the parent ALERT trial outcome, not specifically for the education-mistreatment association. We now present the education analysis as secondary/exploratory and interpret the borderline confidence interval cautiously. Thank you for this important comment. We agree that the original description could be misinterpreted as implying that the sample size was specifically powered for the education–mistreatment association. We have revised the Methods section to clarify that the sample size was determined within the broader ALERT trial framework and was originally based on the ALERT primary perinatal outcome, not on the respectfulness score examined in this cross-sectional analysis. No separate a priori power calculation was conducted for the association between maternal education and reported respectful care. We have therefore interpreted the education finding cautiously, particularly because the adjusted estimate was close to the threshold of statistical significance and the confidence interval was wide. Corrections have been made on lines 147-151.
**Reviewer 1 - Completed rounds per facility:** The implied four to five collection rounds per facility over 27 months are never explicitly stated. The actual number of completed rounds per facility should be reported.	Thank you for this comment. We have revised the Methods section to specify the number of data collection rounds. Data collection was conducted at six months intervals between December 2021 and March 2024. Across this 27-month period, the questionnaire was administered in **five discrete data collection rounds**. The changes were made on lines 139-140.
**Reviewer 1 - Sampling fractions and volume stability:** Three sampling fractions are described by discharge volume, but it is not stated whether volumes were stable over the 27-month period. If higher volumes coincided with intervention phases, sampling fractions could vary systematically between pre- and post-intervention periods, introducing selection bias.	Thank you for this important comment. We have revised the Methods section to clarify that we did not assume discharge volumes were stable across the 27-month study period. Instead, the sampling fraction was determined from the number of eligible women listed for discharge on each data collection day. Thus, the sampling approach was recalibrated daily according to actual discharge volume, rather than being fixed by facility or intervention phase. The same selection rules were applied during both pre- and post-intervention periods: all eligible women were invited when daily discharges were fewer than 14, every second woman was selected when discharges ranged from 20–29, and every third woman was selected when discharges were 30 or more. This approach was intended to maintain feasible and consistent recruitment across facilities and rounds.
**Reviewer 1 - Analytic approach and stepped-wedge structure:** The stepped-wedge structure introduces non-random temporal variation and clustering by facility and time. Standard logistic regression may not account for clustering or time-by-cluster interaction. The intervention period is treated as a binary covariate, which may oversimplify the stepped-wedge exposure structure.	Thank you for this important comment. We agree that the stepped-wedge structure introduces temporal variation and facility-level clustering that should be considered when interpreting the findings. We have revised the manuscript to clarify that this analysis was not designed as an impact evaluation of the ALERT intervention. Rather, it was a secondary cross-sectional analysis examining the association between maternal education and reported mistreatment, with intervention period included as a contextual adjustment variable. We have therefore softened language suggesting an intervention effect and now describe the post-intervention finding as a temporal association.<br>We retained facility as a fixed-effect covariate to account for observed differences between hospitals and adjusted for intervention period to account for broad pre/post differences. However, we acknowledge that treating intervention status as a binary covariate does not fully capture the stepped-wedge exposure structure, secular time trends, or potential facility-by-time interactions. Given the small number of Malawi facilities, more complex mixed-effects or cluster-level models would be statistically unstable.
**Reviewer 1 - Results, education-stratified descriptive findings:** Table 2 presents raw frequencies for mistreatment items across facilities but not by education, the primary exposure. Education-stratified frequencies for key items such as shouting, privacy violations, and non-consented care would strengthen the descriptive results.	Thank you for this helpful comment. We agree that, because education is the primary exposure, the descriptive results should show how key respectful care and mistreatment items vary by education level. We have revised Table 2 to present education-stratified frequencies for each facility and overall, separating women with primary/no education from those with secondary/higher education. This revised table now includes key items such as shouting or screaming by providers, privacy violations, provider permission before vaginal examination, communication, and birth companionship. We have also revised the descriptive results text to highlight the main education-stratified patterns.

Reviewer comment	Response / action taken
**Reviewer 1 - Duplicate reference:** References 12 and 14 appear to be identical (Bohren et al., The Lancet, 2019) and should be corrected.	We thank the reviewer for noting this duplication. References 12 and 14 both cited Bohren et al., *The Lancet* (2019). We have corrected the reference list to remove the duplicate and ensure that each citation is unique and consistently numbered. The revised manuscript now cites Bohren et al. (2019) only once, with numbering adjusted accordingly.
**Reviewer 1 - Flowchart rendering:** The participant flowchart appears to be rendered as plain text rather than as a figure, suggesting a formatting issue in the submitted proof.	Thank you. We have corrected this by submitting the participant flow chart as a high-resolution figure rather than plain text. [Figure 1.]
**Reviewer 1 - Discussion, education-mistreatment interpretation:** The manuscript oscillates between two explanations: education increases awareness/recognition of mistreatment, and education may provoke provider hostility through perceived non-compliance. The authors should attempt to distinguish these empirically, for example by examining whether the education effect differs across mistreatment subtypes.	Thank you for this valuable comment. We revised the Results and Discussion to better distinguish whether higher reporting among women with secondary/higher education reflects greater awareness and recognition of poor care, or differences in provider–patient interactions linked to perceived assertiveness. The Results now highlight education-stratified item-level patterns, including higher reporting of shouting among women with secondary/higher education (lines 244–252), and present sensitivity analyses comparing the broader composite outcome with a narrower abuse-focused measure (lines 282–295). The Discussion now interprets awareness, expectations, reporting thresholds, and provider–patient dynamics as overlapping but distinguishable mechanisms, clarifying that the association may reflect recognition and willingness to report rather than greater exposure to direct abuse alone (lines 322–359).
**Reviewer 1 - Discussion, lower reporting among less-educated women:** The finding that lower reporting among less-educated women may reflect under-recognition rather than lower exposure should be more prominent in the abstract and conclusion. Current framing may be misread as suggesting that more educated women face greater risk.	Thank you for this important comment. We have revised the abstract and conclusion to avoid implying that women with secondary/higher education necessarily experienced greater mistreatment. The abstract now states that higher reporting among women with secondary/higher education may reflect greater recognition, expectations, willingness to report, or provider responses, rather than better care among less-educated women (lines 33–38). The Discussion further clarifies that lower reporting among women with less education may reflect normalisation of poor treatment, limited rights awareness, or fear of questioning providers, rather than better care (lines 384–392). The conclusion has also been revised to frame the education finding cautiously, noting that recognition, expectations, care-seeking patterns, and organisational culture may shape both experiences and reporting (lines 456–469).
**Reviewer 1 - Differential item functioning:** The discussion should engage with measurement literature on differential item functioning by education level. If more educated women interpret identical care behaviours differently, this is a psychometric issue and should be acknowledged as a core limitation.	Thank you for this important comment. We agree that the association between education and poorer respectful care may partly reflect differences in how women interpret and report similar care experiences, rather than differences in exposure alone. We have revised the Discussion to engage with measurement literature on differential item functioning and measurement invariance, noting that education may shape comprehension, expectations, response thresholds, and willingness to classify care as poor (lines 339–351). We also acknowledge this as a core limitation by interpreting education-related differences cautiously and noting that respectful care items, particularly those on communication, dignity, consent, and emotional support, may vary by education-related expectations and reporting thresholds (lines 348–351).
**Reviewer 1 - Facility-level comparison:** Facility 4 is faith-based, charges fees, serves a potentially different socioeconomic population, and has distinct governance and staffing norms. Employment status does not capture wealth, out-of-pocket expenditure, or care-seeking selectivity. Case-mix differences may explain part of the facility effect.	Thank you for this important comment. We agree that the lower odds of reported mistreatment observed in Facility 4 should not be interpreted as a straightforward comparison between faith-based and public facilities. We have revised the Discussion and Limitations sections to acknowledge that Facility 4 differs from the public hospitals in several important ways, including fee-for-service care, governance structure, staffing norms, patient population, delivery volume and caesarean section rate this has been corrected on lines 361-370. Although the adjusted model included employment status as a proxy for socioeconomic position, we recognise that this does not fully capture household wealth, out-of-pocket expenditure, care-seeking preferences, or selective use of faith-based services. We have therefore softened the interpretation of the facility-level finding and clarified that unmeasured socioeconomic and case-mix differences may partly explain the observed lower odds of reported mistreatment at Facility 4.
**Reviewer 1 - Major revision conclusion:** Major revision is recommended, with expectation that outcome operationalisation and stepped-wedge analytic concerns are substantially revised. Without addressing these, the central finding cannot be considered sufficiently supported.	Thank you for this overall assessment; we have substantially revised the manuscript to clarify outcome operationalisation, item harmonisation, the 25th percentile cut-off, sensitivity analyses, and the stepped-wedge implementation schedule, while treating intervention period as a contextual temporal covariate and presenting a more transparent and cautious interpretation of the education–mistreatment association.

Reviewer comment	Response / action taken
**Reviewer 2 - overall recommendation:** The paper's most important contribution is not the prevalence estimate per se, but the operationalisation and application of a composite, multi-domain respectful maternity care index. The paper should be reframed around this methodological contribution rather than primarily as a prevalence study.	Thank you for this helpful comment. We agree that the manuscript needed clearer wording on the scope of the analysis and interpretation of the outcome. However, we respectfully maintain that the study's contribution is not limited to demonstrating the application of a validated multi-domain respectful maternity care index. Rather, the index provides a robust way to examine women's reported experiences of poorer respectful maternity care across multiple domains, including dignity, privacy, communication, social support, hospital environment, and absence of physical and verbal abuse.<br>We have revised the manuscript to make the outcome framing more precise without changing the overall focus of the paper. The abstract, introduction, discussion, and conclusion now clarify that the study applies a validated composite respectful maternity care index to assess women's reported childbirth care experiences across four ALERT-enrolled hospitals in Malawi and to examine variation by maternal education and facility context. We have also revised generalisability statements to clarify that findings apply to the programme-supported hospitals included in this analysis and should not be interpreted as population-level estimates for routine maternity care in Malawi, the Central Region, or nationally. The revised study aim now reflects this framing.
**Reviewer 2 - strengths of data collection:** Structured interviews with 1,004 women across four facilities, a refusal rate of 0.2%, tablet-based REDCap with real-time validation, and systematic random sampling calibrated to discharge volumes represent a high standard of implementation research fieldwork.	Thank you for recognising the strengths of the data collection process.
**Reviewer 2 - scope and generalisability:** This is not a study of the prevalence of mistreatment in Malawi's maternity care system. All four facilities were part of the ALERT quality-improvement programme and are not representative of routine hospital care. The 16% estimate characterises care in supported, actively observed hospitals and should not be generalised to Malawi's Central Region or nationally.	Thank you for this important clarification. We agree that the 16% estimate should not be interpreted as the prevalence of mistreatment in Malawi's maternity care system, the Central Region, or nationally. We have revised the manuscript to clarify that this estimate refers to poorer reported respectful maternity care among women in four ALERT-enrolled hospitals, based on the composite index (lines 27–31). We also reframed the study to emphasise the application of a validated multi-domain respectful maternity care index in programme-supported facilities, rather than presenting it primarily as a prevalence study. These revisions are reflected in the abstract and study framing (lines 13–38), introduction (lines 83–90), Methods description of the composite index and domains (lines 163–183), Strengths and Limitations section (lines 48–51 and 404–415), and conclusion, which highlights the value of composite measures in implementation research (lines 456–469).
**Reviewer 2 - ALERT intervention interpretation:** The paper does not evaluate the ALERT intervention. A credible impact evaluation would require baseline data from the same facilities or a comparable control group. The pre/post binary variable captures a temporal association, not a causal estimate.	Thank you for this important clarification. We agree that this analysis does not evaluate the causal effect of the ALERT intervention. We have revised the manuscript to make this explicit and have revised all language that could imply intervention impact. The pre/post variable is now described as an intervention-period or contextual temporal variable, included to account for broad differences between observations collected before and after the ALERT rollout. We no longer interpret the lower odds of reported mistreatment in the post-intervention period as evidence of intervention effectiveness but instead present it as a temporal association observed within ALERT-enrolled facilities.
**Reviewer 2 - intervention context and null associations:** The intervention context may shape the null results. In quality-improvement facilities where care is monitored and providers are trained, disparities by employment, age, parity, and other sociodemographic characteristics may be attenuated compared with routine care settings.	Thank you for this important observation. We agree that the ALERT quality-improvement context may have influenced the observed null associations for employment, age, parity, and other sociodemographic characteristics. We have revised the Discussion to acknowledge that, because all facilities were participating in a supported implementation programme with provider training, monitoring, mentorship, and quality-

	improvement activities, differences in reported care experiences by some sociodemographic characteristics may have been reduced or less visible than they might be in routine, unsupported maternity care settings.
**Reviewer 2 - concluding scope:** The strongest contribution is the measurement framework and demonstration that a multi-domain respectful maternity care index can be applied consistently across diverse hospital settings. The contribution, scope, and generalisability claims should be revised with more precise language.	Thank you for this helpful comment. We agree that the manuscript needed more precise language on its contribution, scope, and generalisability. We have revised the framing to clarify that a key contribution is the application of a validated multi-domain respectful maternity care index across four ALERT-enrolled hospitals in Malawi. The index provides a standardised way to assess women's reported childbirth care experiences across domains including dignity, privacy, communication, social support, hospital environment, and absence of physical and verbal abuse.<br>However, we respectfully maintain that the study's contribution extends beyond the measurement framework alone. The index also enables examination of how poorer reported respectful maternity care varies by maternal education and facility context. We have therefore revised the abstract, introduction, discussion, and conclusion to clarify the outcome framing and avoid presenting findings as population-level prevalence estimates. We also revised generalisability statements to specify that findings apply to the programme-supported hospitals included in this analysis and should not be interpreted as representative of routine maternity care in Malawi, the Central Region, or nationally.

VERSION 2 - REVIEW

**Reviewer**	**3**
**Name**	**Dack, Kyle**
**Affiliation**	**University of Bristol, MRC Integrative Epidemiology Unit**
**Date**	**25-Aug-2026**
**COI**	

Statistical review:

Overall the paper is well presented, with clear methods and reasonable interpretation. However, I have two concerns regarding (a) the selection of confounders, and (b) the seeming inconsistency between binary and continuous model results. These are commented on in more detail below.

Abstract:

1. "Secondary or higher education was associated with higher odds of reporting poorer respectful maternity care (aOR 1.48, 95% CI 1.01–2.18, p=0.04)" I think it would be more precise and also would ensure readers do not misinterpret this, to explain it is associated with higher odds of being in the bottom 25% of care scores.

Methods:

2. Sample size - It is reasonable as a secondary analysis that the N is determined by the parent study. However, line 153 is missing a fullstop.

3. DAG: It is a strength that the study used a DAG to determine potential confounders. However, there are pathways which are not consistent with the descriptions:

-Intervention period: This is reported in the manuscript as a potential confounder, but in the DAG it is shown as a mediator (pathway: education > intervention period > mistreatment). I am unsure how either is possible. Based on the manuscript, intervention period was adjusted for separate reasons, which makes me think it should not be part of the DAG.

-Employment: This is labelled as (employment > education) but described as (employment > education).

-Facility: This is labelled as (facility > education) but described as (education > facility).

-Parity: As above, arrow is (parity > education) but described as (education > parity).

If the descriptions for employment, facility, and parity are accurate, then these arrows should all be reversed. This will make them mediators and they probably should not be adjusted for, because adjustment may split the association of interest. I think the authors used Daggity, and this will automatically show the updated confounder variables, if the arrows are corrected.

Based on the descriptions, I think that age may be the only true confounder in the DAG, as it is the only one which could affect both the outcome and education status.

I recommend investigating this because it could improve the accuracy of the model estimates, and reduce the uncertainty in the 95% CI.

Results:

4. " Descriptive patterns of education" - it would be extremely helpful to also report the mean, SD, and median of the outcome score, stratified by education. This would help to understand the inconsistency which I comment on below.

Additionally, this table is missing a number.

5. Table 4 / continuous sensitivity analysis. It is reported that " Across different model specifications, the findings were largely consistent". But by my interpretation, I do not think these findings are consistent with the main result.

The continuous linear regression result shows that higher education score correlate with better care, albeit overlapping with the null (b = 1.03). The main binary result shows the opposite, higher education is correlated with higher odds of being in the bottom 25% of scores (aOR 1.48).

So the main model shows an inverse correlation between education / outcome, and the continuous model shows a positive correlation.

Statistically, this is possible. It suggests the distribution and skew of the outcome in the two education categories could be different. The descriptive statistics are not reported for me to fully evaluate this, but it could be such as:

-There are 20% of people in the higher education group with a care score less than 84.5, and a mean score of 90.

-There are 14% in the primary education group with less than 84.5, and a mean score of 87.

It would be helpful to investigate this, by looking at the descriptive statistics and distribution (e.g. histograms) of the outcome per education group. I suggest also acknowledging this inconsistency in the discussion, and offering explanation. It would also be good to perform model diagnostic checks on the linear continuous model, and report whether the assumptions were met.

6. Table 4: There are three other models reported in this table but these were not in the methods. Please add and clarify their purpose.

VERSION 2 - AUTHOR RESPONSE

**Reviewer 3**	
1: In the Abstract, Secondary or higher education was associated with higher odds of reporting poorer respectful maternity care (aOR 1.48, 95% CI 1.01–2.18, p=0.04)" I think it would be more precise and also would ensure readers do not misinterpret this, to explain it is associated with higher odds of being in the bottom 25% of care scores.	We thank the reviewer for this helpful suggestion. We agree that the original wording could be misinterpreted, as it does not make clear that the outcome refers to being in the lowest-scoring group for respectful maternity care rather than a general measure of poor care. We have revised the sentence in the Abstract on page 1 to clarify this point.
2: Sample size - It is reasonable as a secondary analysis that the N is determined by the parent study. However, line 153 is missing a full stop.	Thank you. We have added the missing full stop on page5 and carefully reviewed the surrounding text for similar punctuation errors.
3a: DAG: It is a strength that the study used a DAG to determine potential confounders. However, there are pathways which are not consistent with the descriptions: -Intervention period: This is reported in the manuscript as a potential confounder, but in the DAG it is shown as a mediator (pathway: education > intervention period > mistreatment). I am unsure how either is	We thank the reviewer for carefully reviewing the DAG and identifying inconsistencies between the arrows and the descriptions in the manuscript. We agree that the DAG required revision and have corrected the causal pathways and adjustment set accordingly.<br>3a. Intervention period: We agree that intervention period should not be included in the DAG. It was not considered a confounder of the relationship between education and mistreatment. Rather, it

possible. Based on the manuscript, intervention period was adjusted for separate reasons, which makes me think it should not be part of the DAG.	was included in the regression model to account for the stepped-wedge ALERT study design and changes over calendar time. We have therefore removed intervention period from the DAG and clarified in the Methods that it was adjusted for because of the stepped-wedge design, rather than as a confounder identified by the DAG.<br>Supplemental file 2
3b. Employment, facility and parity: The arrows for employment, facility and parity appear to be inconsistent with their descriptions. If the descriptions are correct, these arrows should be reversed, potentially making these variables mediators rather than confounders (Employment: This is labelled as (employment > education) but described as (employment > education).<br>3c**:** Based on the descriptions, age may be the only true confounder because it could affect both education and the outcome. The authors are encouraged to investigate this, as it may improve the accuracy of the estimates and reduce uncertainty in the confidence intervals (Facility: This is labelled as (facility > education) but described as (education > facility).	We agree that the original DAG contained inconsistencies between the direction of the arrows and the descriptions in the manuscript. We have corrected the DAG to reflect the proposed causal relationships. Specifically, education is shown as influencing employment, facility and parity, which in turn may influence mistreatment. These variables are therefore considered potential mediators rather than confounders and have been removed from the adjustment set. Age is shown as a common cause of both education and mistreatment and is therefore retained as the confounder in the adjusted model. Following these corrections, we re-ran the DAG in DAGitty to identify the appropriate adjustment set. The revised DAG indicates that age is the only variable that needs to be adjusted for to estimate the association between education and mistreatment. We therefore revised the adjusted regression models to adjust for age only and removed employment, facility and parity from the adjustment set.<br>Revisions have been made on pages 7 and 13.
If the descriptions for employment, facility, and parity are accurate, then these arrows should all be reversed. This will make them mediators and they probably should not be adjusted for, because adjustment may split the association of interest. I think the authors used Daggity, and this will automatically show the updated confounder variables, if the arrows are corrected.	We have updated the DAG (Supplemental File 2), the Analysis subsection of the Methods, and the corresponding regression results throughout the manuscript to reflect the revised causal model and adjustment strategy. We thank the reviewer for identifying this issue, which has helped us improve the clarity and consistency of our causal analysis.
4: Under " Descriptive patterns of education" - it would be extremely helpful to also report the mean, SD, and median of the outcome score, stratified by education. This would help to understand the inconsistency which I comment on below. Additionally, this table is missing a number.	Thank you for this helpful suggestion. We have included Table 3 on page12 to report the mean (SD) and median (IQR) of the outcome score, stratified by education group, both overall and by facility. These descriptive statistics provide greater clarity on the distribution of the outcome score across education groups and help contextualise the observed differences in the regression analyses. We have also corrected the missing number in table 2.

5: Table 4 / continuous sensitivity analysis. It is reported that " Across different model specifications, the findings were largely consistent". But by my interpretation, I do not think these findings are consistent with the main result. The continuous linear regression result shows that higher education score correlate with better care, albeit overlapping with the null (b = 1.03). The main binary result shows the opposite, higher education is correlated with higher odds of being in the bottom 25% of scores (aOR 1.48).<br><br>So the main model shows an inverse correlation between education / outcome, and the continuous model shows a positive correlation.	Thank you for raising this point. In Table 5, in order to make it easier for the reader, the continuous score was flipped so that higher scores meant less respectful care (for the purpose of being comparable with the other sensitivity analyses where the outcome was less respectful care), thus the continuous linear regression result showed that higher education is correlated with a lower respectful maternity care score, which was consistent with the binary analysis. However, we see now that this could be unclear and confusing, thus we have changed back to the original continuous score in Table 5. Using the original respectful maternity care score, where higher scores mean more respectful care, the coefficient was negative (β = -1.06, 95%CI -2.11 to - 0.01, p=0.05), which is consistent with the other models.
5b**:** Statistically, this is possible. It suggests the distribution and skew of the outcome in the two education categories could be different. The descriptive statistics are not reported for me to fully evaluate this, but it could be such as:<br>-There are 20% of people in the higher education group with a care score less than 84.5, and a mean score of 90.<br>-There are 14% in the primary education group with less than 84.5, and a mean score of 87.	In Table 5, the continuous score was flipped so that higher scores meant less respectful care. We see now that this could be confusing, thus we now use the original continuous score in Table 5. The continuous linear regression shows that higher education correlate with a lower respectful maternity care score (β = -1.06, 95%CI -2.11 to - 0.01, p=0.05).
5c: It would be helpful to investigate this, by looking at the descriptive statistics and distribution (e.g. histograms) of the outcome per education group. It would also be good to perform model diagnostic checks on the linear continuous model, and report whether the assumptions were met.	Thank you for this helpful suggestion. We have now examined the distribution of the continuous respectful maternity care (RMC) score by education group using descriptive statistics. The descriptive results show that the distribution of RMC scores was broadly similar between women with primary/no education and those with secondary/higher education, although the groups differed somewhat in their mean and median scores. These distributions are now presented in the revised manuscript (Table 3) on page 12.
5d: The inconsistency between the binary and continuous models should be acknowledged and discussed, with a possible explanation.	The direction of the binary and continuous models were consistent. We have revised the table to make this clearer.
6: Table 4 reports three additional models that were not described in the Methods. Please add these models to the Methods and clarify their purpose.	We thank the reviewer for this observation. We agree that the additional models presented in Table 4 were not adequately described in the Methods, and we have revised the Analysis section to include

	and explain each of them. Revisions have been made on page 7
**Reviewer 3 – Competing interests**	**Response:** Noted. No competing interests were reported.

VERSION 3 - REVIEW

**Reviewer**	**3**
**Name**	**Dack, Kyle**
**Affiliation**	**University of Bristol, MRC Integrative Epidemiology Unit**
**Date**	**29-Sep-2026**
**COI**	

I appreciate the authors responses which addressed all my queries. However, as a minor comment regarding formatting, Line 170 is still missing a full stop.
