# Supplementary material for: Respectful care during childbirth and educational inequities: a cross-sectional survey using a multidomain index across four hospitals in Malawi
Source: BMJ Open. 2026 Oct 21;16(10):e118574. doi: 10.1136/bmjopen-2026-118574 (PMC13691797; doi:10.1136/bmjopen-2026-118574)
Supplement: Supplementary file 4 [file bmjopen-16-10-s003.docx]

**Supplemental Table 3. Data collection rounds and facility intervention status**

| **Data collection round** | **Data collection dates** | **Facility 1: Mchinji Hospital** | **Facility 2: Ntcheu District Hospital** | **Facility 3: Mitundu Rural Hospital** | **Facility 4: St Gabriel’s Hospital** |
| --- | --- | --- | --- | --- | --- |
| Round 1 | December 2021 | Control | Control | Control | Control |
| Round 2 | August–September 2022 | Intervention | Control | Control | Control |
| Round 3 | February–March 2023 | Intervention | Intervention | Control | Control |
| Round 4 | August 2023 | Intervention | Intervention | Intervention | Control |
| Round 5 | February 2024 | Intervention | Intervention | Intervention | Intervention |
