# Supplementary material for: Respectful care during childbirth and educational inequities: a cross-sectional survey using a multidomain index across four hospitals in Malawi
Source: BMJ Open. 2026 Oct 21;16(10):e118574. doi: 10.1136/bmjopen-2026-118574 (PMC13691797; doi:10.1136/bmjopen-2026-118574)
Supplement: Supplementary file 6 [file bmjopen-16-10-s005.docx]

**Completed CROSS Checklist for Reporting of Survey Studies**

Manuscript: Mistreatment During Childbirth and Educational Inequities: A Cross-sectional Study Across Four Hospitals in Malawi

**Note:** Page and line numbers are based on the current line-numbered manuscript draft. They should be checked once the final revised manuscript is paginated by the journal system.

| **Section/topic** | **Item** | **CROSS item description** | **Reported on page/line #** |
| --- | --- | --- | --- |
| Title and abstract | 1a | State the word “survey” along with a commonly used term in the title or abstract to introduce the study design. | p.1, title lines 1–2 |
| Title and abstract | 1b | Provide an informative summary in the abstract covering background, objectives, methods, findings/results, interpretation/discussion, and conclusions. | pp.1–2, abstract lines 10–39 |
| Introduction: Background | 2 | Provide a background about the rationale of the study, what has been previously done, and why this survey is needed. | pp.2–3, lines 52–91 |
| Introduction: Purpose/aim | 3 | Identify specific purposes, aims, goals, or objectives of the study. | p.3, lines 87–91 |
| Methods: Study design | 4 | Specify the study design in the Methods section with a commonly used term, such as cross-sectional or longitudinal. | p.3, lines 93–99 |
| Methods: Data collection methods | 5a | Describe the questionnaire, including number of sections, number of questions, and names of instruments used. | p.4, lines 112–130 |
| Methods: Data collection methods | 5b | Describe questionnaire instruments used to measure concepts, target population, validity/reliability information, scoring/classification procedure, and references. | p.4, lines 112–130; pp.5–6, lines 168–200 |
| Methods: Data collection methods | 5c | Provide information on pretesting of the questionnaire, if performed. | p.4, lines 119–123 and 132–139 |
| Methods: Data collection methods | 5d | Provide the questionnaire in the article, appendix, or online supplement, if possible. | p.4, lines 128–130; p.6, lines 191–194; Supplementary File 2 |
| Methods: Sample characteristics | 6a | Describe the study population, locations, eligibility criteria, and exclusion criteria. | pp.3–5, lines 100–110 and 159–166; p.7, lines 239–240 |
| Methods: Sample characteristics | 6b | Describe the sampling techniques used and specify participant locations where clustered sampling was applied. | pp.4–5, lines 140–151 |
| Methods: Sample characteristics | 6c | Provide information on sample size and sample size calculation. | p.5, lines 152–158 |
| Methods: Sample characteristics | 6d | Describe how representative the sample is of the study or target population, particularly for population-based surveys. | p.2, lines 50–51; p.18, lines 424–435 |
| Methods: Survey administration | 7a | Provide information on questionnaire administration, including type and number of contacts and survey location. | pp.4–5, lines 132–139 and 159–166 |
| Methods: Survey administration | 7b | Provide the survey time frame, including recruitment, exposure, and follow-up periods. | pp.4–5, lines 140–151 |
| Methods: Survey administration | 7c | Describe the entry process and approaches used to minimise data entry error for non-web surveys or prevent multiple participation for web surveys. | p.4, lines 132–139; p.5, lines 164–166 |
| Methods: Study preparation | 8 | Describe preparation before conducting the survey, such as interviewer training or advertising. | p.4, lines 119–123 and 132–139 |
| Methods: Ethical considerations | 9a | Provide information on ethical approval, informed consent, institutional review board approval, and relevant declarations. | p.26, lines 753–758 |
| Methods: Ethical considerations | 9b | Provide information about anonymity/confidentiality and mechanisms used to protect against unauthorised access. | p.26, lines 759–763; p.7, lines 236–238 |
| Methods: Statistical analysis | 10a | Describe statistical methods and analytical approach and report the statistical software used. | pp.6–7, lines 211–230 |
| Methods: Statistical analysis | 10b | Report any modification of variables used in the analysis, with reference if available. | pp.5–6, lines 168–210 |
| Methods: Statistical analysis | 10c | Report details on how missing data were handled, including missing items and methods used. | p.7, lines 228–230; p.9, lines 245–246 |
| Methods: Statistical analysis | 10d | State how non-response error was addressed. | p.7, lines 239–240; Figure 1, p.8 |
| Methods: Statistical analysis | 10e | For longitudinal surveys, state how loss to follow-up was addressed. | N/A |
| Methods: Statistical analysis | 10f | Indicate whether weighting, propensity scores, or other methods were used to adjust for non-representativeness. | N/A |
| Methods: Statistical analysis | 10g | Describe any sensitivity analysis conducted. | p.6, lines 220–222; p.14, line 298; p.15, lines 303–313 |
| Results: Respondent characteristics | 11a | Report numbers of individuals at each stage of the study and consider using a flow diagram. | p.7, lines 239–240; Figure 1, p.8, lines 241–243 |
| Results: Respondent characteristics | 11b | Provide reasons for non-participation at each stage, if possible. | p.7, lines 239–240; Figure 1, p.8 |
| Results: Respondent characteristics | 11c | Report response rate and define the response rate or formula used. | p.7, lines 239–240; response rate formula not explicitly stated |
| Results: Respondent characteristics | 11d | For web-based surveys, explain how unique visitors were determined and report visitor proportions. | N/A |
| Results: Descriptive results | 12 | Provide participant characteristics and information on potential confounders and assessed outcomes. | pp.8–9, Table 1 and lines 244–257; pp.10–12, Table 2; p.13, lines 260–268 |
| Results: Main findings | 13a | Give unadjusted and, where applicable, confounder-adjusted estimates with 95% confidence intervals and p-values. | pp.13–14, Table 3 and lines 275–296 |
| Results: Main findings | 13b | For multivariable analysis, provide model-building process, model fit statistics, and model assumptions as appropriate. | p.6, lines 215–220; p.7, lines 228–230; Supplementary Files 3–4 |
| Results: Main findings | 13c | Provide details of any sensitivity analysis performed, including complete-case versus imputed analyses if relevant. | p.6, lines 220–222; p.14, line 298; p.15, lines 297–312 |
| Discussion: Limitations | 14 | Discuss limitations, including potential biases and imprecision, such as non-representativeness, study design, and uncontrolled confounding. | p.18, lines 412–434; p.2, lines 48–51 |
| Discussion: Interpretations | 15 | Give a cautious overall interpretation based on biases and imprecision and suggest areas for future research. | pp.14–16, lines 313–411; p.17 lines 455–468 |
| Discussion: Generalizability | 16 | Discuss external validity of the results. | p.2, lines 50–51; p.18, lines 424–435 |
| Other sections: Role of funding source | 17 | State whether funders had any role in survey design, implementation, and analysis. | pp.25, lines 766–769; funder role in design, implementation, and analysis not explicitly stated |
| Other sections: Conflict of interest | 18 | Declare any potential conflict of interest. | p.25, lines 770–771 |
| Other sections: Acknowledgements | 19 | Provide names of organisations/persons acknowledged and their contribution. | Not currently reported |

**Checklist source:** Consensus-Based Checklist for Reporting of Survey Studies (CROSS).
