# Supplementary material for: Respectful care during childbirth and educational inequities: a cross-sectional survey using a multidomain index across four hospitals in Malawi
Source: BMJ Open. 2026 Oct 21;16(10):e118574. doi: 10.1136/bmjopen-2026-118574 (PMC13691797; doi:10.1136/bmjopen-2026-118574)
Supplement: Supplementary file 7 [file bmjopen-16-10-s002.docx]

**Supplemental File 2: Revised Directed Acyclic Graph (DAG)**
